# Molecular mechanisms of traditional Chinese medicine in skin aging: a narrative review

**DOI:** 10.3389/fphar.2026.1792159

**Published:** 2026-08-19

**Authors:** Sisi Yin, Huilan Zheng, Qingjing Yang, Xingyu Chen, Qianqian Yang, Qifeng Yang, Hongbin Cheng, Jingping Wu

**Affiliations:** 1 Department of Medical Cosmetology, Hospital of Chengdu University of Traditional Chinese Medicine, Chengdu University of Traditional Chinese Medicine, Chengdu, Sichuan, China; 2 School of Clinical Medicine, Chengdu University of Traditional Chinese Medicine, Chengdu, Sichuan, China; 3 Department of Dermatology, Hospital of Chengdu University of Traditional Chinese Medicine, Chengdu University of Traditional Chinese Medicine, Chengdu, Sichuan, China

**Keywords:** anti-aging, cellular senescence, oxidative stress, photoaging, skin aging, traditional Chinese medicine

## Abstract

Skin aging involves complex endogenous and extrinsic mechanisms, driving oxidative stress, cellular senescence, DNA damage, and extracellular matrix degradation. This narrative review synthesizes preclinical evidence on traditional Chinese medicine (TCM)-derived botanical drugs and formulae targeting these pathways. We identified 19 single-botanical drug and 17 formula studies. The most consistently reported effects include Nrf2/ARE activation, reduced reactive oxygen species (ROS) and malondialdehyde (MDA), increased superoxide dismutase (SOD) activity, matrix metalloproteinase (MMP) inhibition, and lower senescence markers [p16, p21, and senescence-associated β-galactosidase (SA-β-Gal)]. Additional modulated pathways include MAPK/AP-1, PI3K/Akt/mTOR, TGF-β/Smad, and NAD+/Sirtuin, collectively contributing to reduced inflammation, enhanced antioxidant defense, and preserved dermal collagen. These findings suggest multi-pathway modulation rather than true pharmacological synergy. This system-level approach highlights a shift from monotherapy toward holistic intervention. However, clinical evidence remains extremely limited and consists mainly of small human studies, including trials described as randomized or double-blind but incompletely reported, and single-arm evaluations; no adequately powered, preregistered, high-quality randomized controlled trial (RCT) has yet established clinical efficacy for TCM interventions in skin aging. Therefore, future research should prioritize rigorous RCTs, dose–response designs, and phytochemical standardization.

## 1 Introduction

Aging is a natural biological process that has been extensively studied for decades. Projections indicate that by 2050, individuals aged 65 years and older will comprise 16% of the global population (Chen C. et al., 2023), with this proportion expected to reach 26.1% in China (Wang H. et al., 2024). This demographic shift poses challenges to healthcare systems worldwide. Among visible signs of aging, skin aging is the most apparent. As the body’s primary interface with the environment, skin aging results from both intrinsic (chronological) and extrinsic (mainly photoaging) factors (Uitto, 2008). Contemporary anti-aging strategies aim to promote skin health and rejuvenation (Griffiths et al., 2023; Goodman and Bagatin, 2024), using topical antioxidants, photoelectric technologies, and injectable therapies (Shin et al., 2019; Zhang et al., 2024). However, these approaches can be costly, may have side effects, and often provide limited long-term benefits. For instance, energy-based technologies improve skin texture through controlled thermal damage (Jia and Feng, 2024), but excessive exposure risks tissue injury. Therefore, safer, more durable, and personalized anti-aging interventions remain a priority.

Traditional Chinese medicine (TCM) offers a distinct approach with multi-component, multi-mechanism therapies (Li D. et al., 2023). By considering individual physiological characteristics, TCM holds a unique position in anti-aging research (Ding et al., 2024). Current investigations include clinical trials evaluating TCM therapies, such as moxibustion, for age-related conditions (Sun et al., 2024). A growing body of experimental evidence suggests that many TCM-derived botanical drugs and formulae can mitigate skin aging by targeting cellular senescence, oxidative stress, and inflammation (Hartinger et al., 2024). Emerging evidence demonstrates that medicine-food homologous plants can improve the endogenous features of skin aging—chronic inflammation, metabolic dysregulation, and impaired repair capacity (Zhu et al., 2025). The theoretical foundations of TCM, detailed in texts such as the “Inner Canon of the Yellow Emperor,” describe aging processes and propose herbal remedies to maintain vitality. Modern research is increasingly focused on elucidating the molecular mechanisms behind these effects (Zhao and Luo, 2017; Zhao et al., 2020). However, most evidence is preclinical (*in vitro* and *in vivo*). Clinical studies are few, small, and methodologically weak. Specifically, the available clinical evidence consists mainly of small human studies, including trials described as randomized or double-blind but incompletely reported, and single-arm evaluations; no adequately powered, preregistered, high-quality randomized controlled trial (RCT) has yet established clinical efficacy for TCM interventions in skin aging. In this review, we therefore focus on molecular mechanisms from preclinical models, with the clinical evidence gap as a key limitation.

We searched PubMed and Web of Science from inception to 6 April 2026, using the search string: ((skin OR cutaneous OR dermal OR epidermal) AND (aging OR ageing OR senescence OR photoaging)) AND (“traditional Chinese medicine*” OR “Chinese herb*” OR “Chinese herbal medicine” OR “botanical drug*”). The search was limited to English articles; reference lists of relevant papers were also screened. We included original *in vitro*, *in vivo*, or human studies on TCM-derived botanical drugs, extracts, or formulae that reported at least one molecular endpoint related to oxidative stress, extracellular matrix (ECM) remodeling, cellular senescence, DNA damage, or inflammation. Reviews, conference abstracts, case reports, and studies on synthetic compounds or non-skin aging models were excluded. The search yielded 427 records (Supplementary Figure S1). After screening, 36 studies were included (19 single botanical drugs and 17 formulae). As this is a narrative review, no formal quality assessment or risk-of-bias scoring was performed.

## 2 Key molecular mechanisms of skin aging

**FIGURE 1:**
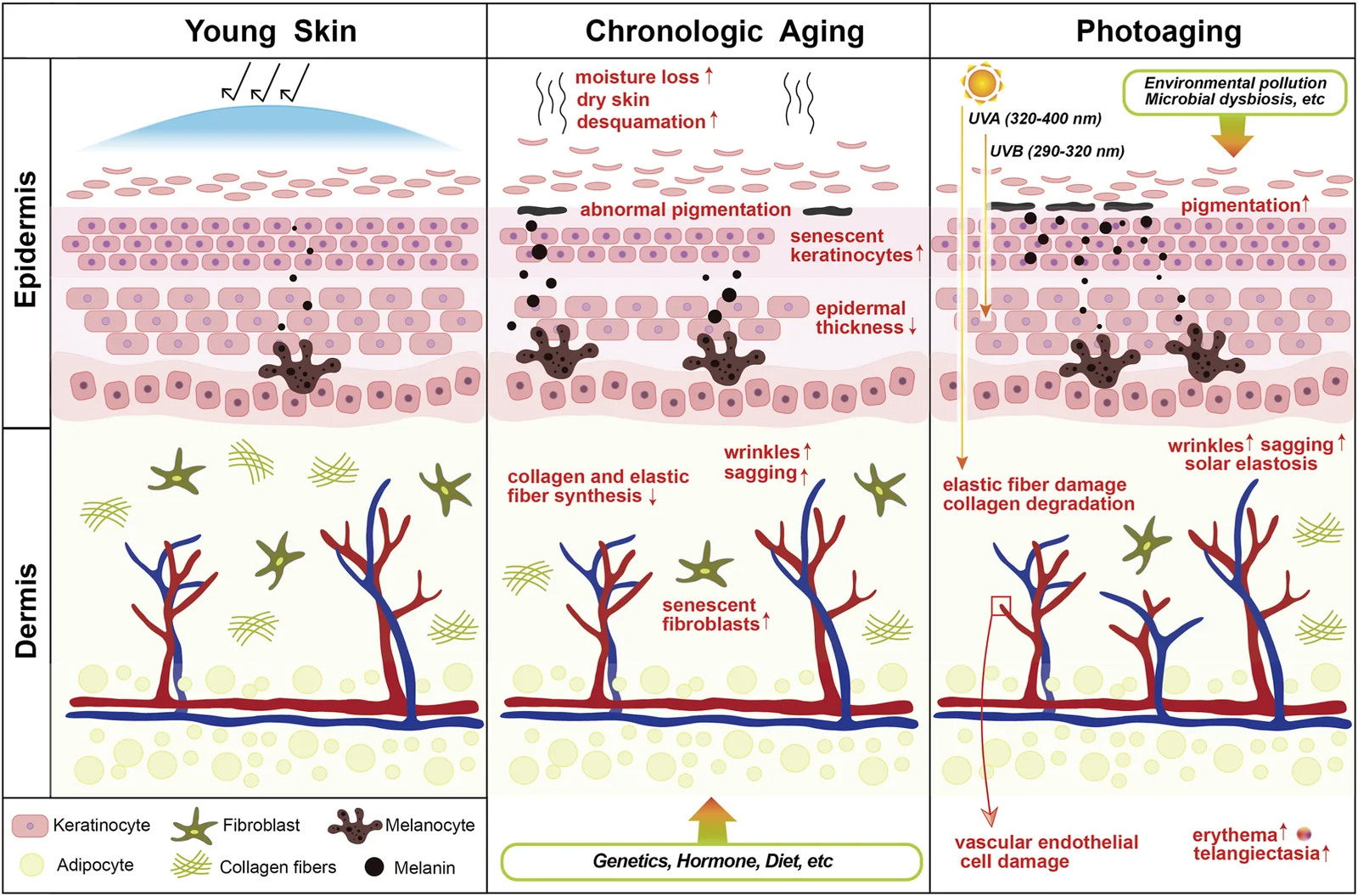
Structural alterations in skin layers during chronological aging and photoaging. This schematic compares the morphology of young skin with that affected by chronological (intrinsic) and photo- (extrinsic) aging. Young skin shows an intact epidermal barrier, a dense dermal extracellular matrix, and normal microvasculature. Chronological aging (driven by genetics and hormones) is characterized by epidermal thinning, reduced collagen/elastin synthesis, and fine wrinkles. Photoaging (primarily caused by UV radiation) leads to epidermal dysplasia, abnormal pigmentation, severe collagen/elastin degradation (solar elastosis), and vascular damage (erythema and telangiectasia). Coarse wrinkles and significant laxity are prominent. Indeed, skin aging involves the convergence of both pathways.

**FIGURE 2:**
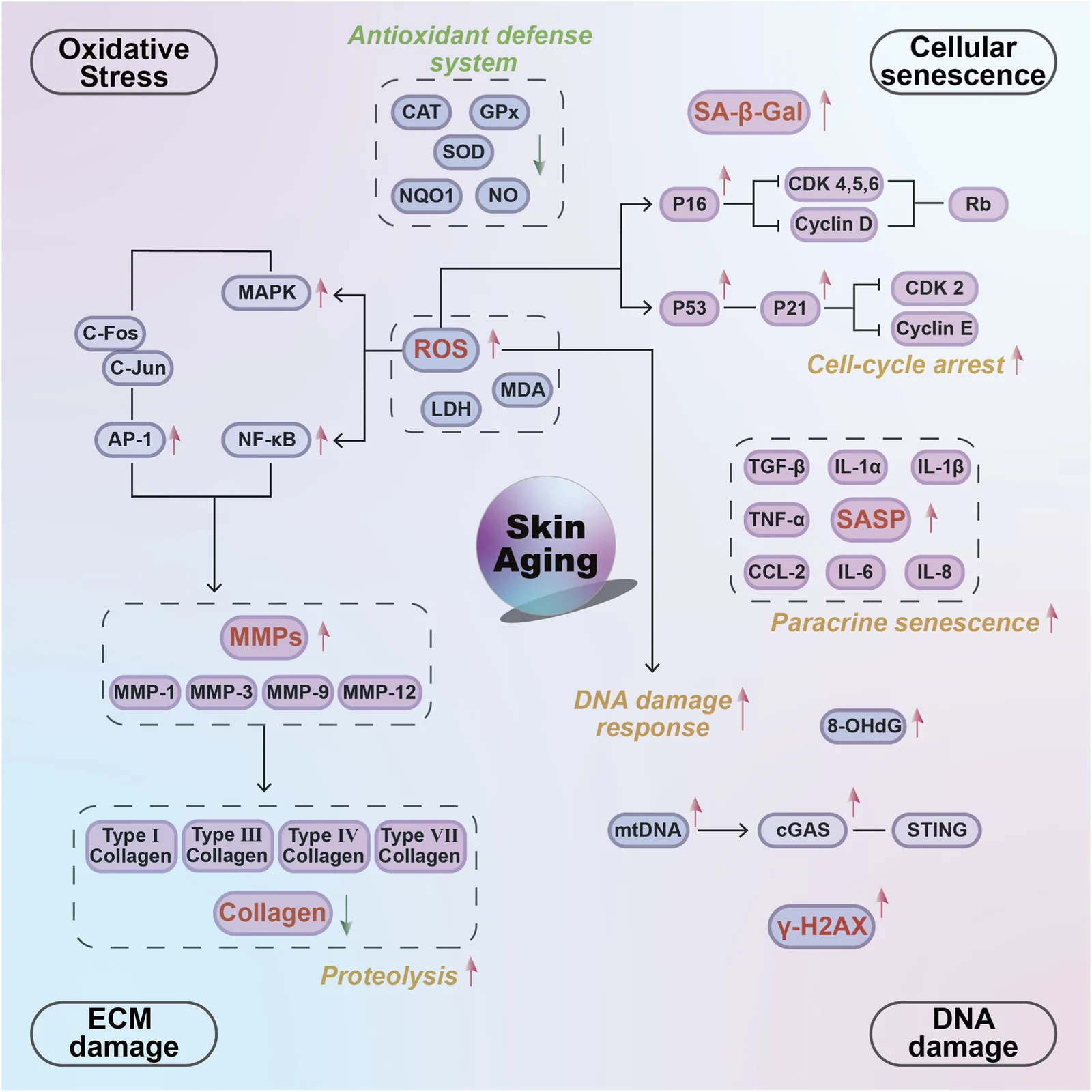
Schematic summary of the key molecular mechanisms driving skin aging. The diagram illustrates the interconnected pathways central to skin aging. Oxidative stress arises from an imbalance between reactive oxygen species (ROS) production and the antioxidant defense system (e.g., SOD and CAT), leading to lipid peroxidation and macromolecular damage. Cellular senescence is characterized by irreversible cell cycle arrest, mediated by upregulation of p16/p21 and CDK inhibitors, and the secretion of pro-inflammatory SASP factors (e.g., IL-6, IL-8, and MMPs). DNA damage, primarily induced by UV radiation, triggers repair responses marked by γH2AX and activates the cGAS–STING pathway, promoting inflammation and apoptosis. Extracellular matrix (ECM) degradation results from the imbalance between collagen synthesis and enzymatic breakdown by MMPs, which are upregulated by ROS and inflammatory signals. These core processes form a reinforcing network that perpetuates the aging phenotype.

Skin consists of epidermis, dermis, subcutaneous tissue, and appendages. Intrinsic aging leads to gradual thinning of the epidermis (Arnal-Forné et al., 2024) and progressive loss of dermal collagen and elastin (Yu et al., 2024), while extrinsic aging—driven mainly by ultraviolet radiation (Araviiskaia et al., 2019; Faria and Andrade, 2024)—accelerates these changes through oxidative stress, inflammation, and matrix degradation (Parkinson et al., 2015) (Figure 1). Both processes are clinically intertwined, but photoaging dominates visible skin aging (Kammeyer and Luiten, 2015). In this review, we focus on four core mechanisms that drive the skin aging phenotype: oxidative stress, cellular senescence, ECM degradation, and DNA damage (Figure 2).

### 2.1 Oxidative stress

Excess reactive oxygen species (ROS) is a primary driver of photoaging (Harman, 1956; Kammeyer and Luiten, 2015; Rinnerthaler et al., 2015). ROS, including superoxide anion (O_2_^−^), hydrogen peroxide (H_2_O_2_), hydroxyl radical (HO·), and singlet oxygen species (^1^O_2_) (Cardoso et al., 2023), damages intracellular components, leading to cell dysfunction and senescence (Gu et al., 2020; Huang and Li, 2020). Biomarkers include malondialdehyde (MDA, lipid peroxidation) (Williams et al., 2014), lactate dehydrogenase (LDH, membrane damage) (Yun et al., 2008; Livesey et al., 2020), and 8-hydroxy-2′-deoxyguanosine (8-OHdG, oxidative DNA damage) (Lee et al., 2013). The skin’s endogenous antioxidant system includes enzymes such as superoxide dismutase (SOD), catalase (CAT), and glutathione peroxidase (GSH-Px), as well as non-enzymatic molecules such as vitamins and glutathione (Michalak, 2022). SOD protects against UV-induced damage (Holley et al., 2012; Holley et al., 2014; Chen D. et al., 2023) and has clinical potential (Dong et al., 2023). The transcription factor Nrf2 controls the expression of these enzymes; its nuclear translocation and activation of the antioxidant response element (ARE) are central to redox homeostasis (Bellezza et al., 2018; Ulasov et al., 2022). Nitric oxide (NO) also has antioxidant and anti-inflammatory properties (Mraz Robinson et al., 2023; Seabra et al., 2023), with post-UV release peaking at approximately 48 h (Hazell et al., 2022), and plasma-derived NO shows anti-aging effects (Jaiswal et al., 2024). Lighter skin may experience prolonged oxidative stress (Tsuchida et al., 2023), and aged skin has a blunted stress response (Dai et al., 2023).

### 2.2 Cellular senescence

Cellular senescence is an irreversible cell cycle arrest, typically triggered by telomere shortening, DNA damage, or oncogenic stress. In the skin, senescent fibroblasts and keratinocytes accumulate over time, especially after exposure to UV radiation. Photoaging induces senescence more effectively than chronological aging (Yu et al., 2024). Oxidative stress accelerates telomere shortening and DNA damage, thereby creating a vicious cycle (von Zglinicki, 2000; Rezvan, 2017; Cavinato et al., 2024). The senescence-associated secretory phenotype (SASP) includes pro-inflammatory cytokines such as IL-6, IL-1β, and TNF-α, chemokines, and enzymes that degrade cellular matrices such as matrix metalloproteinase 1 (MMP-1) and MMP-3. SASP exacerbates senescence in the cells that produce it and spreads it to neighboring cells through paracrine signaling (Acosta et al., 2013; Kumari and Jat, 2021; Mebratu et al., 2023; Wang B. et al., 2024). Common biomarkers of senescence include senescence-associated β-galactosidase (SA-β-Gal) (Lee et al., 2006; Debacq-Chainiaux et al., 2009; Bulbiankova et al., 2023) and cyclin-dependent kinase inhibitors such as p16^INK4a^ and p21^CIP1/WAF1^ (Di Cunto et al., 1998; Huang et al., 2022; Cohn et al., 2023). Increased expression of p16^INK4a^ is associated with damage to dermal elastic fibers (Waaijer et al., 2016). Although senescence has tumor-suppressive effects, its chronic activation contributes to aging and inflammation (Takeuchi et al., 2010; Huang et al., 2022). Neutrophils and the NLRP1 inflammasome, *via* the protein gasdermin D, can also contribute to the process of senescence (Awad et al., 2018; Muela-Zarzuela et al., 2024; Xu Z. et al., 2024).

### 2.3 Extracellular matrix degradation

The dermal ECM is primarily composed of type I and III collagen and elastin (Liu et al., 2024). Age-related decreases in collagen levels are due to reduced synthesis by fibroblasts and increased degradation by MMPs, particularly MMP-1, MMP-3, and MMP-9 (Pittayapruek et al., 2016; Lee et al., 2021). UV exposure stimulates the activity of MMPs through ROS-dependent activation of MAPK/AP-1 and NF-κB pathways, resulting in collagen breakdown, wrinkling, and loss of skin firmness (Fisher et al., 1998; Hwang et al., 2019). TNF-α and UVB-induced DNA damage also increase MMP expression (Ågren et al., 2015; Kim et al., 2022). MMP-1 itself can accelerate fibroblast senescence (Quan et al., 2023). Collagens IV, VII, and XVII also decrease with age (Langton et al., 2016). Deficiencies in NRF2 or reduced mitochondrial mass impede collagen production (Katsuyama et al., 2022; Salamito et al., 2023).

### 2.4 DNA damage and repair

UV radiation induces the formation of cyclobutane pyrimidine dimers (CPDs) and other types of photoinduced DNA damage (Nakanishi et al., 2009; Schuch et al., 2017). Blue light also damages DNA in keratinocytes (Chamayou-Robert et al., 2022). If these damaged areas are not repaired, they can lead to mutations, cell death, or cellular senescence. The DNA damage marker γH2AX increases with age in skin and is associated with the process of senescence (Mah et al., 2010; Zorin et al., 2019). Aging slows the formation of γH2AX foci and impairs the ability of cells to repair double-strand DNA breaks (Oizumi et al., 2024; Suzuki et al., 2024). Cells rely on nucleotide excision repair to maintain genomic stability (Kemp et al., 2017). The cGAS–STING pathway detects DNA fragments in the cytoplasm, such as those resulting from mitochondrial DNA damage after exposure to UVB radiation (Li C. et al., 2023). This pathway activates NF-κB, leading to inflammation and cell death (Li et al., 2021). Impaired DNA repair accelerates the aging process, as observed in progeroid syndromes.

### 2.5 Mechanisms of skin aging models

Three experimental systems are commonly used in skin aging research: naturally aged animals, D-galactose-induced accelerated aging, and UV-induced photoaging. In this review, we categorize skin aging into two broad types—intrinsic (chronological) aging and extrinsic (photo)aging—and therefore, all three models can be discussed under the unifying theme of “skin aging.” However, it is important to recognize that their underlying pathological mechanisms differ substantially, and these differences should be considered when interpreting intervention outcomes.

Naturally, aged animals exhibit progressive intrinsic decline characterized by chronic low-grade inflammation, metabolic dysregulation, diminished repair capacity, and gradual ECM degradation. Replicative senescence models, which mimic intrinsic aging through serial passaging, have been shown to most closely approximate the transcriptomic features of human chronological aging (Fang et al., 2025). In contrast, the UV-induced model represents extrinsic photoaging, driven by acute DNA photodamage, an immediate burst of reactive oxygen species, strong inflammatory responses (high SASP), and rapid MMP-mediated collagen breakdown—pathways that differ markedly from intrinsic aging in both tempo and mechanism. The D-galactose model occupies a different but highly instructive position: excess D-galactose triggers oxidative stress, accumulation of advanced glycation end-products (AGEs), mitochondrial dysfunction, and chronic low-grade inflammation *via* NF-κB and MAPK activation, thereby recapitulating the core features of intrinsic skin aging—dermal thinning, collagen loss, diminished elasticity, and impaired repair capacity—in an accelerated manner (Umbayev et al., 2020).

Despite these mechanistic distinctions, dermal fibroblast senescence emerges as a common driver across all three models. The SASP, mitochondrial dysfunction, and persistent activation of pathways such as NF-κB, p16/pRB, mTOR, and TGF-β collectively perpetuate ECM degradation and chronic inflammation (Nan et al., 2025).

## 3 Single botanical drugs and extracts against skin aging

We included 19 original studies on TCM-derived botanical drugs or natural extracts for skin aging. Detailed pharmacological parameters for each study, including extract type, analytical characterization, dose/concentration ranges, treatment duration, and positive/negative controls, are provided in Supplementary Table S1. Their evidence levels ranged from II (single-arm clinical trial or incomplete RCT reporting) to IV (*in vitro* only), as classified in Table 1. Three studies were classified as Level II: *Cordyceps sinensis* (Berk.) Sacc. (Cordycipitaceae) (incomplete RCT reporting), *Morus alba* L. (Moraceae) root (incomplete RCT reporting), and *Paeonia suffruticosa* Andr. (Paeoniaceae) (single-arm trial). The remaining 16 studies were classified as level III (animal with *in vitro*) or level IV (*in vitro* only). These studies focused primarily on oxidative stress, extracellular matrix degradation, cellular senescence, and related signaling pathways (Nrf2/ARE, MAPK/AP-1, PI3K/Akt/mTOR, TGF-β/Smad, NAD+/Sirtuin, *etc.*), which align with the four mechanisms outlined above.

**TABLE 1:** Summary of TCM-derived extracts and their molecular mechanisms against skin aging.

No.	First author, year, doi	TCM/Extract	Core compounds (analytical method)	Oxidative stress	DNA damage	Cellular senescence/SASP/inflammation	ECM degradation	Molecular expression/signaling pathway	Experimental models (*In vitro*/*in vivo*/clinical)	Evidence level
1	Guanghui Sun, 2022, 10.3389/fphar.2022.991917	*Scutellaria baicalensis* Georgi (Lamiaceae) (root, 80% ethanol reflux)	Baicalin, baicalein, wogonoside, and wogonin (LC–MS/MS)	↑SOD, ↓ROS, and ↓MDA	↓p53	↓SA-β-gal	↑hydroxyproline, ↑collagen, and ↓MMP1	Antagonizes REV-ERBα → upregulates BMAL1	*In vitro*: NIH3T3, HaCaT, mouse fibroblasts, human keratinocytes (D-gal/UVB, 24 h); *in vivo*: male C57BL/6J mice (D-gal + UVB, topical, 25–400 mg/kg, n = 7); clinical: none	III
2	Kunting Zhao, 2026, 10.3390/ijms27052304	*Cardiospermum halicacabum* L. (Sapindaceae) (whole plant, 70% ethanol)	Myricitrin, luteolin, apigenin, rutin, and kaempferol (UPLC–Orbitrap–MS and HPLC)	↓ROS	↓p-p53/p53 and ↓p16	↓p16, ↓p-p53, and relieve S-phase arrest	Not detected	↓p-PI3K/PI3K, ↓p-Akt/Akt, and ↓p-mTOR/mTOR (inhibits PI3K/Akt/mTOR)	*In vitro*: human skin fibroblasts (UVB, 25–100 μg/mL, 24 h); *in vivo*: none; clinical: none	IV
3	Li Shao, 2023, 10.1371/journal.pone.0274479	*Cordyceps cicadae* (Miq.) Massee (Cordycipitaceae) (fruiting body, hot water)	Polysaccharides, mannitol, nucleosides, and polyphenols (HPLC and HPAEC)	ABTS/DPPH scavenging and FRAP	Not detected	Not detected	↓MMP12 and ↑hyaluronic acid	↑HAS2, ↑HBEGF, ↑CCL2, and ↑IL6; ↓MMP12	*In vitro*: human skin fibroblasts (50–100 μg/mL, 24–48 h); *in vivo*: none; clinical: none	IV
4	ZhengWang Sun, 2018, 10.1002/ptr.6100	*Paeonia suffruticosa* Andr. (Paeoniaceae) (root, 70% ethanol)	Paeonol (HPLC)	↓ROS, ↑Nrf2 nuclear, ↑HO-1, and ↑NQO-1	Not detected	Not detected	↓MMP-1 and ↑procollagen I	↓p-ERK, ↓p-JNK, ↓p-p38, ↓p-c-fos, ↓p-c-jun; ↑DLD, and ↑Nrf2 nuclear	*In vitro*: HaCaT (UVB, 1–10 μg/mL, 24–72 h); *in vivo*: female HR-1 hairless mice (UVB, topical, PA 0.1%/1%, n = 8); clinical: none	III
5	Junxi Liu, 2025, 10.3389/fphar.2025.1520392	*Paeonia suffruticosa* Andr. (Paeoniaceae) (root bark, ethanol + AB-8 resin)	Paeonol, penta-O-galloyl-β-D-glucose (HPLC)	↓ROS, ↑SOD2, ↑CAT, and ↑Nrf2	↓ATM and ↓GADD45A	↓SA-β-gal	↑Col I/III/IV and ↓MMP1	↓p-IRS1, ↓p-PI3K, ↓p-AKT, and ↓mTOR; ↑p-FOXO and ↑Nrf2 nuclear	*In vitro*: HaCaT (UVB), HFF (UVA), 20–40 μg/mL; *in vivo*: none; clinical: single-arm, n = healthy adults 30–50years (TEWL, hydration and elasticity)	II
6	Shishu Xu, 2024, 10.1016/j.jep.2024.118352	*Chrysanthemum × morifolium* (Ramat.) Hemsl. (Asteraceae) (flower bud, water + D101 resin)	4,5-Dicaffeoylquinic acid, 3,4-dicaffeoylquinic acid, and luteolin-7-O-glucoside (HPLC–Q-TOF–MS/MS)	↓ROS, ↑GSH, ↑Nrf2 nuclear, ↑HO-1, and ↑NQO1	Not detected	Not detected	↓MMP1 and ↑collagen fiber	↓p-p38, ↓p-JNK, and ↓p-ERK; ↑Nrf2 nuclear, ↑HO-1, and ↑NQO1 (MAPK and Nrf2/ARE)	*In vitro*: HaCaT, HFF-1 (UVB, 25–100 μg/mL, 24 h); *in vivo*: female ICR mice (UVB, topical, 0.208/0.417 mg/cm2/d, n = 6); clinical: none	III
7	Xin Yan, 2025, 10.3389/fphar.2025.1692491	*Oryza sativa* L. (Poaceae) [rice bran, 50% ethanol + *Aspergillus oryzae* (Ahlb.) Cohn (Trichocomaceae) fermentation]	Azelaic acid, ferulic acid, γ-tocotrienols, δ-tocotrienols, and squalene (UPLC–Q-TOF–MS/MS)	Not detected	Not detected	Not detected	↑Col I, ↑Col III, ↑elastin, and ↑collagen deposition	↑Col I/III mRNA; no specific pathway reported	*In vitro*: mouse skin fibroblasts (2D/3D, 1–10 μg/mL); *in vivo*: male Balb/c mice (normal skin, topical, 14/28days, n = 3–6); clinical: none	III
8	Chien-Liang Chao, 2023, 10.3390/life13112130	*Poria cocos* (Schw.) Wolf (Polyporaceae) (sclerotium, 75% ethanol, and lanostane triterpenoids)	Pachymic acid, dehydropachymic acid, tumulosic acid, etc. (UPLC, NMR, and ESI–MS)	Not detected	Not detected	Not detected	↑Col I and ↑hyaluronic acid	↑Col I protein and ↑hyaluronic acid production; no pathway reported	*In vitro*: primary human dermal fibroblasts (0.01–1 μM, 24–48 h); *in vivo*: male SD rats (D-gal, intragastric, 1–6 mg/kg, n = 6); clinical: none	III
9	Yafan Yang, 2015, 10.1155/2015/619,560	*Taraxacum officinale* F.H.Wigg. (Asteraceae) (leaf/flower/root, water)	Not reported	↓ROS and ↑GSH	Not detected	↓SA-β-gal (H2O2-induced)	↓MMP activity	↑Glutathione reductase (GR) mRNA	*In vitro*: human dermal fibroblasts (UVB/H2O2, 30–300 μg/mL, 24–72 h); *in vivo*: none; clinical: none	IV
10	Mengyang Zhang, 2018, 10.1089/rej.2017.1971	*Prunella vulgaris* L. (Lamiaceae) (flower spike and 50% ethanol)	Rosmarinic acid, caffeic acid, and rutin (HPLC)	↓ROS and DPPH scavenging	Not detected	↓IL-6 and ↓TNF-α	↓MMP-1, ↓MMP-3, and ↑procollagen I	↓p-ERK, ↓p-JNK, ↓p-c-Fos, ↓p-c-Jun, ↓NF-κB p65; ↑TGF-β1, ↑p-Smad2/3, and ↓Smad7	*In vitro*: normal human dermal fibroblasts (UVB, 1–100 μg/mL, 24–72 h); *in vivo*: none; clinical: none	IV
11	Wenyuan Chen, 2024, 10.3389/fphar.2024.1431391	*Hibiscus mutabilis* L. (Malvaceae) (leaf, 50% ethanol + resin)	Kaempferol, quercetin, morin, naringenin, and scrophulein (UPLC–MS/MS)	Not detected	Not detected	↓TNF-α, ↓IL-6, and ↓IL-17	↓MMP-9 and ↑collagen density	↓p-AKT and ↓p-STAT3 (inhibits AKT/STAT3)	*In vitro*: HaCaT (UVB + UVA, 125–500 μg/mL, 2 h); *in vivo*: male ICR mice (UV, topical, 200/400 mg/kg, n = 6); clinical: none	III
12	Ling Xiao, 2024, 10.3892/etm.2024.12411	*Crocus sativus* L. (Iridaceae) (stigma, aqueous)	Crocin, picrocrocin (HPLC)	↑SOD and ↓MDA	Not detected	↓TNF-α, ↓IL-1β, ↑IL-4, and ↑IL-10	↑epidermal/dermal thickness	↑NAMPT (brain), ↑NAD+ (brain); regulates NAMPT/NAD+	*In vitro*: none; *in vivo*: female C57BL/6J mice (natural aging, intragastric, 80 mg/kg, n = 8); clinical: none	III
13	Ritamaria Di Lorenzo, 2024, 10.3390/ijms25084282	*Cordyceps sinensis* (Berk.) Sacc. (Cordycipitaceae) (mycelium and 50% hydroethanol)	Cordycepin, adenosine (UHPLC–DAD)	↓cytosolic ROS	Not detected	Not detected	↑Pro-collagen I	↑SIRT1, ↑SIRT3, ↑SIRT6, ↑NAD+, and ↑ATP	*In vitro*: HaCaT, HDFs (0.0006% C. sinensis extract, 6/24 h); *in vivo*: none; clinical: RCT, double-blind, placebo-controlled, n = 40 (skin energy, collagen, and wrinkle)	II
14	Guang Sun, 2023, 10.1016/j.jep.2022.115883	*Panax ginseng* C.A.Mey. (Araliaceae) (cv. Silvatica) (root, water + D101 resin)	7 phenolic acids (protocatechuic, p-hydroxybenzoic, chlorogenic, vanillic, caffeic, salicylic, and ferulic) (HPLC–DAD)	↓ROS, ↓MDA, ↑SOD, ↑CAT, ↑GPx, ↑GSH, and ↑Nrf2 nuclear	↓8-OHdG	↓SA-β-gal, ↓p53, ↓p21, and ↓p16	↑Col I	↓Keap1 and ↑Nrf2 nuclear (Nrf2/ARE)	*In vitro*: HFF-1 (UVA, 6.25–25 μg/mL, 24 h); *in vivo*: none; clinical: none	IV
15	Xue-Lin Chen, 2022, 10.1016/j.toxicon.2022.106934	*Kochia scoparia* (L.) Schrad. (Amaranthaceae) (dried fruit and water decoction)	Total phenolics, flavonoids (no specific monomers)	↑DPPH/ABTS scavenging and ↑FRAP	↓AAPH-induced DNA oxidative damage	Not detected	Not detected	Not reported	*In vitro*: none; *in vivo*: KM mice and SD rats (acute/subacute toxicity, intragastric, 2.5–15 g/kg); clinical: none	III
16	Mengzhu Liu, 2026, 10.1186/s13020-026-01344-w	*Dracaena cochinchinensis* (Lour.) S.C.Chen (Asparagaceae) (stem resin, raw extract in micelle hydrogel)	Loureirin A/B, 7,4′-dihydroxyflavone, resveratrol, pterostilbene (LC-QqQ-MS/MS)	Improved redox balance (metabolomics)	Not detected	↓SA-β-gal (HDFs)	↑collagen density, ↑elastic fiber, and ↓epidermal hyperplasia	Normalized purine, arginine-NO, TCA; activated Nrf2/HO-1, inhibited NF-κB/MAPK	*In vitro*: HDFs (UV); *in vivo*: female SKH-1 hairless mice (UV, topical, 0.25%/1.0% RD, n = 8); clinical: none	III
17	Hanane Chajra, 2020, 10.3390/molecules25184162	*Morus alba* L. (Moraceae) (root, ethanol + 1,3-propanediol/water)	Moracenin A/B, Kuwanon C, Wittiorumin F, and Morusin (UHPLC–MS/MS and NMR)	Not detected	Not detected	Not detected	↓MMP-1, ↓CCN1, ↑COL3A1, ↑procollagen I, and ↓collagenase activity	↓MMP-1, ↓CCN1, and ↑COL3A1; inhibited collagenase	*In vitro*: NHDFs (UVB, 0.00625%–0.2%); *in vivo*: none; clinical: RCT, double-blind, placebo-controlled, n = 22 female (wrinkle depth, smoothness, and plumping)	II
18	Yi-Chen Li, 2013, 10.3109/13880209.2012.721130	*Hyriopsis cumingii* Lea (Unionidae) (pearl, room temp supercritical water)	Water-soluble proteins, amino acids, Ca2+, Mg2+ (not specified)	Not detected	Not detected	Not detected	↑Col III mRNA	↑Col III/I mRNA ratio; no pathway reported	*In vitro*: Hs68, HFF primary fibroblasts (scratch wound, 300 μg/mL, 16 h); *in vivo*: none; clinical: none	IV
19	Zhongmin Zhang, 2022, 10.1002/cbdv.202100876	*Pinctada fucata martensii* (pearl, water extract + hydrogel)	14 amino acids (HPLC–PITC)	↑SOD, ↑CAT, ↑GSH-Px, and ↓MDA	Not detected	↓IL-1β, ↓IL-6, ↓TNF-α, ↓COX-2, and ↓PGE2	↓MMP-1, ↓MMP-3, and ↑hydroxyproline (collagen)	No specific pathway; inhibited MMPs and enhanced antioxidant enzymes	*In vitro*: none; *in vivo*: female KM mice (UV, topical, 0.2–0.8 mg/mouse, n = 9); clinical: none	III

Evidence level definition:

• I–High-quality randomized controlled trial (RCT, double-blind, placebo-controlled, adequate sample size, and predefined endpoints).

• II–Clinical trial with incomplete reporting (e.g., missing registration number, primary endpoint, or randomization details), non-randomized clinical trial, or single-arm clinical study (human data without placebo control or blinding).

• III–*In vivo* animal study (with or without *in vitro* cell data; includes efficacy endpoints in animal models).

• IV–*In vitro* cell study only (molecular mechanism studies without animal or clinical validation).

Abbreviations: ↑/↓ indicate increase or decrease of the parameter; “Not detected” means that the study did not report or examine that phenotype.

### 3.1 Antioxidant effects and Nrf2 signaling regulation

Antioxidative activity is the most extensively studied direction in TCM research on skin aging. Of the studies in this category, the highest evidence level is level II. No level I human data support Nrf2-mediated antioxidant effects for any single botanical drug against skin aging.

*Scutellaria baicalensis* Georgi (Lamiaceae) (Sun et al., 2022) (level III) was confirmed to alleviate skin aging induced by D-galactose combined with UVB, reduce wrinkles, increase SOD and hydroxyproline (HYP), inhibit the expression/activity of MMP-1/p53, and upregulate BMAL1 in a dose-dependentmanner by antagonizing REV-ERBα. Previous studies have demonstrated that BMAL1 inhibits *Propionibacterium acnes*-induced skin inflammation through its downstream target REV-ERBα *via* repressing the NF-κB/NLRP3 signaling axis, further supporting the critical role of the REV-ERBα/BMAL1 axis in skin homeostasis (Li F. et al., 2022). Sun et al. (2022) introduced the circadian clock REV-ERBα/BMAL1 axis into the field of TCM against skin aging. However, only the total extract was tested, and the bioactive compounds acting as the REV-ERBα antagonist were not identified, which represents a major limitation.

UPLC–Orbitrap–MS revealed that *Cardiospermum halicacabum* L. (Sapindaceae) extract (Zhao et al., 2026) (level IV) is rich in myricitrin, luteolin, and other flavonoids. In UVB-irradiated fibroblasts, it was shown to scavenge ROS, improve mitochondrial dysfunction, alleviate S-phase cell cycle arrest, and inhibit overactivation of the PI3K/Akt/mTOR pathway. The integration of transcriptomics in this study is a major highlight, but it remains at the *in vitro* cellular level, and no pathway rescue validation was conducted, making the mechanistic evidence correlative rather than causal.

*Cordyceps cicadae* (Miq.) Massee (Cordycipitaceae) aqueous extract (Shao et al., 2023) (level IV) demonstrated ABTS/DPPH-free radical scavenging and ferric ion reduction capabilities. In human skin fibroblasts, it promoted hyaluronic acid synthesis by upregulating HAS2, HBEGF, CCL2, and IL6 and downregulating MMP12. Mechanistic validation was limited to transcriptomics and RT-PCR; no protein-level (Western blot) confirmation was provided. The study focuses solely on hydration, lacks animal and human data, and remains at the *in vitro* proof-of-concept stage. As level IV evidence, its translational value is minimal.

In terms of deeper mechanisms, subsequent studies have explored the regulatory effects on Nrf2 signaling. The root extract of *P. suffruticosa* Andr. (Paeoniaceae) (Sun et al., 2018) (level III), which contained paeonol as the main compound and was confirmed to downregulate AP-1 activation by blocking ERK/JNK/p38 phosphorylation while upregulating DLD expression, Nrf2 nuclear translocation, and HO-1/NQO-1 expression. This study elucidates a dual-pathway mechanism of paeonol against photoaging involving DLD/Nrf2/ARE and MAPK/AP-1. However, only high and low doses were used, and reverse validation through DLD or Nrf2 knockout was not performed, resulting in incomplete mechanistic verification.

Another study on *P. suffruticosa* Andr. (Paeoniaceae) root extract (Liu et al., 2025) (level II) identified penta-O-galloyl-β-D-glucose as an additional active compound. It demonstrated through HaCaT cells, HFF cells, and a reconstructed human full T-Skin™ model that it reduces UV-induced ROS, SA-β-gal, and MMP1; upregulates SOD2, CAT, and Nrf2; inhibits overactivation of IRS1/PI3K/AKT/mTOR; promotes FOXO phosphorylation; and repairs collagen structure. Clinically, it was shown to reduce transepidermal water loss (TEWL), increase hydration and elasticity, and improve roughness. In this study, a closed-loop validation approach encompassing bioactive compounds, *in vitro* cell models, 3D skin tissues, and clinical trials was established.

The extract of buds of *Chrysanthemum × morifolium* (Ramat.) Hemsl. (Asteraceae) (Xu S. et al., 2024) (level III) contains 16 polyphenolic compounds (4 phenolic acids and 12 flavonoids) and was validated in HaCaT/HFF-1 cells and ICR mice to inhibit phosphorylation of MAPK (p38/JNK/ERK) and activate Nrf2 nuclear translocation and downstream HO-1/NQO1 expression. Notably, the use of UVB-irradiated HFF cells is methodologically questionable as fibroblasts are typically damaged by UVA rather than UVB. Validation by pathway knockout, silencing, or core monomer dose–effect analysis is lacking. A common limitation of the above studies is that whether Nrf2 is a necessary mediator remains unproven at the genetic level.

### 3.2 Extracellular matrix regulation

ECM metabolic imbalance is among the core phenotypes of skin aging. Of the studies, most are classified as level III or level IV with significant design flaws. No human clinical data (Level II or higher) support ECM-related effects for single botanicals. In terms of promoting ECM synthesis, *C. cicadae* (Miq.) Massee (Cordycipitaceae) aqueous extract has been previously shown to increase hyaluronic acid synthesis *via* HAS2.

Fermented rice bran extract (Yan et al., 2025) (Level III) employs *Aspergillus oryzae* (Ahlb.) Cohn (Trichocomaceae) fermentation combined with 50% ethanol extraction. UPLC–QTOF–MS/MS revealed eight bioactive compounds, including azelaic acid and ferulic acid. In 2D/3D fibroblast models and mice, it significantly promoted the synthesis of type I/III collagen and elastin, reduced TEWL, and increased skin tensile strength. The methodological strengths of this study include the combination of fermentation processes with 3D skin models, but dose-dependent validation of multiple key indicators remains insufficient. The multi-pathway mechanisms of core compounds have not been elucidated, and most critically, the use of normal healthy mice rather than aging/photoaging models weakens the specificity of its antiaging conclusions.

*Poria cocos* (Schw.) Wolf (Polyporaceae) lanostane triterpenoid extract (Chao et al., 2023) (level III) dose-dependently restored skin thickness and increased type I collagen and hyaluronic acid levels in D-galactose-induced aging rats. *In vitro* experiments confirmed that triterpenoid compounds such as pachymic acid are antiaging substances that promote collagen and hyaluronic acid synthesis. However, we did not explore upstream signaling pathways or detect changes in aging biomarkers, resulting in insufficient mechanistic depth.

In terms of inhibiting ECM degradation, *Taraxacum officinale* F.H.Wigg. (Asteraceae) extract (Yang and Li, 2015) (level IV) inhibited MMP overactivation, reduced ROS generation, increased GSH levels and glutathione reductase (GR) mRNA expression, and increased UVB absorption capacity in fibroblasts, whereas the root extract had significantly weaker effects. This study clarifies the differences in antiphotoaging efficacy among different parts of dandelion, but it is limited to *in vitro* experiments, lacks identification of bioactive compounds, and uses only a single high dose, resulting in insufficient robustness of the mechanism.

*Prunella vulgaris* L. (Lamiaceae) extract (Zhang et al., 2018) (level IV) exhibited ROS-scavenging activity, downregulated MMP-1/3 and IL-6/TNF-α, upregulated of type I procollagen, and mechanistically inhibited ERK/JNK phosphorylation and the NF-κB p65 pathway while activating the TGF-β/Smad2/3 pathway and suppressing Smad7. This study covers four aspects—anti-inflammatory, antioxidant, anti-degradation, and pro-synthesis—but it is limited to *in vitro* cell experiments and does not clarify the specific contributions of bioactive compounds, leaving the causal structure of its molecular mechanism network unclear.

Analysis of the mechanisms underlying the ability of *Hibiscus mutabilis* L. (Malvaceae) extract (Chen et al., 2024) (level III) to alleviate UV-induced skin photoaging through enhanced network pharmacology and experimental validation identified 22 bioactive compounds using UPLC‒MS/MS; the authors screened targets such as AKT1, STAT3, and MMP9 *via* network pharmacology and confirmed *in vivo* and *in vitro* that it can block overactivation of the AKT/STAT3 pathway and inhibit MMP9 overexpression. Although the network pharmacology analysis in this study provides a multitarget hypothesis, the lack of gene knockdown/rescue validation means that the “multi-component, multi-target” concept remains primarily based on associations.

### 3.3 Cellular senescence and DNA damage

Of the studies in this category, one is classified as level II (*C. sinensis* (Berk.) Sacc. (Cordycipitaceae)) with incomplete RCT reporting, which receives proportionally greater analytical attention here. The remaining studies are classified as level III or IV. Some studies have directly targeted the molecular mechanisms of cellular senescence markers and DNA damage repair.

*Crocus sativus* L. (Iridaceae) aqueous extract (Xiao et al., 2024) (level III) was administered via gavage for 8 weeks in naturally aged mice, which increased epidermal and dermal thickness in the skin and elevated NAMPT and NAD^+^ levels in the brain. We focus on the antiaging mechanism of saffron on the NAMPT/NAD+ axis in natural aging mice, but the severe lack of molecular marker data for skin aging raises concerns about the skin specificity of the conclusions.

In contrast, *C. sinensis* (Berk.) Sacc. (Cordycipitaceae) mycelium extract (Di Lorenzo et al., 2024) (level II) upregulated SIRT1/SIRT3/SIRT6 in HaCaT cells *in vitro*, increased NAD^+^ and ATP, scavenged ROS, and promoted type I procollagen synthesis in fibroblasts. The study claims to be a randomized, double-blind, placebo-controlled clinical trial (28 days of topical application), showing a 52% increase in skin energy, 10% increase in collagen, and 28% reduction in wrinkles. However, the reporting is incomplete: no trial registration number, no primary endpoint specification, and limited details on randomization and blinding. Therefore, despite the RCT design, it is classified as level II due to reporting deficiencies. Limitations also include exclusive female subjects, short duration (28 days), and lack of animal safety data. Long-term safety and transdermal mechanisms remain unclear.

*Panax ginseng* C.A.Mey. (Araliaceae) (cv. Silvatica) (Sun et al., 2023) (level IV) contains seven phenolic acids. The phenolic acid fraction dose-dependently reduced 8-OHdG levels, reduced the percentage of SA-β-gal-positive cells, and decreased p53/p21/p16 protein expression in UVA-damaged HFF-1 cells by promoting Nrf2 nuclear translocation through Keap1 inhibition. This study explicitly focuses on protection against DNA oxidative damage, but it is only an *in vitro* study, lacks Nrf2 validation experiments, and does not analyze the optimal ratio or synergistic contributions of the seven phenolic acids.

Additionally, the water decoction of *Kochia scoparia* (L.) Schrad. (Amaranthaceae) Fructus (Chen et al., 2022) (level III) was used to evaluate its antioxidant and antiaging potential and oral safety. The ethyl acetate fraction protects against AAPH-induced DNA and protein oxidative damage. Acute and 28-day subacute toxicity experiments confirmed no mortality at 15 g/kg, with 5 g/kg being the no-observed-adverse-effect level. The toxicological evidence from this study is its unique contribution. However, the core antioxidant monomers were not identified, skin-related *in vitro* experiments were not conducted, and only healthy animals were used; thus, the evidence for the anti-skin-aging efficacy of this material remains indirect.

### 3.4 Formulation innovation and drug delivery technology

The studies in this category are classified as levels III and II [*M. alba* L. (Moraceae), incomplete RCT reporting], respectively.

The *Dracaena cochinchinensis* (Lour.) S.C.Chen (Asparagaceae) resin (Resina Draconis) micelle hydrogel (Liu et al., 2026) (level III) is the most complex formulation design among the groups in the literature. In this study, Resina Draconis was loaded into poloxamer micelles and prepared into a hydrogel, achieving an encapsulation efficiency of 80%–90% for five compounds, namely, loureirin A, loureirin B, 7,4′-dihydroxyflavone, resveratrol, and pterostilbene, with approximately 80% release over 24 h following Higuchi kinetics. It demonstrated efficient transdermal delivery and targeted deposition in the epidermis and dermis. In UV-induced photoaging mice, it significantly improved collagen loss and elastic fiber degeneration. Nontargeted metabolomics revealed its ability to reverse purine, arginine, and other metabolic disorders. The integration of nanodrug delivery and metabolomics in this study represents the modernization direction of TCM. However, the absence of core target knockdown/rescue validation, unclear individual compound contributions, exclusive use of female mice, and lack of human clinical data leave a significant gap in the “mechanistically targeted” claim at the target validation level.

*Morus alba* L. (Moraceae) root extract (Chajra et al., 2020) (level II) used aeroponic plant milking technology combined with nitrogen deficiency induction, increasing prenylated flavonoid content by 18-fold compared with that of commercial products. *In vitro*, it inhibited collagenase (73% inhibition rate), downregulated MMP-1 and CCN1, and upregulated COL3A1. A clinical trial described as double-blind and placebo-controlled showed reduced wrinkle depth and improved skin smoothness and plumpness. However, the reporting is incomplete: the sample size is small (n = 22 female), no trial registration number is provided, and key details on randomization, primary endpoint, and blinding methods are missing. Therefore, despite the RCT design, it is classified as level II. The non-destructive, sustainable production technology is innovative, but the lack of target validation leaves the “multi-pathway anti-aging” mechanism unconfirmed at the combination of bioactive compounds.

Pearl extraction has been reported in two studies. In the first study (Li et al., 2013) (level IV), the results demonstrated that 300 μg/mL pearl water extract increased the number of scratch-migrated cells by approximately 3-fold and significantly upregulated collagen type III mRNA expression relative to type I. This study focused on the cell migration-promoting and wound repair activities of pearls, but it evaluated only a single concentration, did not identify bioactive compounds, and did not explore signaling pathways. The second study (Zhang et al., 2022) (level III) prepared a pearl extract hydrogel and demonstrated that it dose-dependently improved wrinkles and sagging, enhanced SOD/CAT/GSH-Px activity, reduced MDA and IL-1β/IL-6/TNF-α levels, and inhibited MMP-1/3, with high-dose effects significantly superior to those of vitamin E. This study confirmed the triple mechanisms of antioxidant, anti-inflammatory, and antidegradation activities but did not identify bioactive compounds, validate the signaling pathway, or provide sufficient evidence on action targets and translational outcomes.

### 3.5 Common methodological deficiencies and overall assessment

In 19 studies, the following common high-frequency deficiencies can be summarized: (1) inadequate compound identification and quality control—most studies report only total flavonoid/phenolic content or chromatographic peak assignments, with only a few studies providing more detailed identification; (2) lack of systematic dose design—the vast majority of animal experiments use only a single dose or two high/low doses, without dose‒response and time‒effect relationship studies, making it difficult to distinguish specific regulation from nonspecific stress responses; (3) widespread sex bias—among the nine studies that explicitly reported animal sex, five used only female and four used only male subjects; extrapolating conclusions from a single sex to the entire population is common practice in these studies; (4) absence of reverse genetic validation—except for the *S. baicalensis* Georgi (Lamiaceae) study that performed REV-ERBα knockout, all other pathway studies failed to conduct rescue experiments after gene knockdown/knockout.

## 4 Traditional Chinese formulae for skin aging

This section includes 17 studies on TCM compound formulations for anti-skin aging (Table 2). Detailed composition, preparation methods, and quality control information for each formula are presented in Supplementary Table S2, while detailed pharmacological parameters are summarized in Supplementary Table S3. In terms of research approaches, network pharmacology, combined with molecular docking and experimental validation, has become a mainstream strategy (adopted by more than half of the studies), but the evidence hierarchy remains dominated by *in vitro* cell experiments and *in vivo* animal models. Only the Bazishen capsule provided single-arm clinical trial data. From a mechanistic depth perspective, these studies focus primarily on the Nrf2/ARE antioxidant pathway, MAPK/PI3K–Akt signaling axis, apoptosis‒autophagy balance, p53/p21/p16 senescence markers, and ECM metabolic regulation, which align closely with the core framework of this review. In the following sections, level III studies are discussed as animal-level evidence requiring further validation in humans, while level IV studies are explicitly noted as preliminary, hypothesis-generating *in vitro* findings.

**TABLE 2:** Summary of TCM formulas and their molecular mechanisms against skin aging.

No.	First author, year, doi	TCM formula	Core compounds (analytical method)	Oxidative stress	DNA damage	Cellular senescence/SASP/inflammation	ECM degradation	Molecular expression/signaling pathway	Experimental models (*In vitro*/*In vivo*/clinical)	Evidence level
1	Tao Liu, 2023, 10.1016/j.jep.2022.115935	Erzhi formula (80% ethanol)	Salidroside, specnuezhenide, isoquercitrin, echinacoside, verbascoside, isochlorogenic acid A, and wedelolactone (UPLC)	↓ROS, ↓MDA, ↑SOD, ↑CAT, ↑GSH-Px, and ↑Nrf2 nuclear	↓DNA damage (comet assay)	↓apoptosis, ↓LDH, and ↑Bcl-2/Bax	Not detected	↑Nrf2 nuclear, ↑HO-1, and ↑NQO1 (Nrf2/HO-1/NQO1)	*In vitro*: HaCaT (UVB, 10−3–10–1 mg/mL); *in vivo*: male ICR mice (UVA + UVB, topical, 0.67/1.33 mg/g, n = 6); clinical: none	III
2	Shan Zhu, 2022, 10.3389/fphar.2022.976473	Modified Qing’e formula (75% ethanol)	Salvianic acid A, chlorogenic acid, rosmarinic acid, salvianolic acid B, psoralen, corylin, cryptotanshinone, and tanshinone IIA (UHPLC–Q-TOF–MS)	↓ROS, ↓MDA, ↑SOD, ↑CAT, ↑GSH-Px, and ↑Nrf2 nuclear	Not detected	↓apoptosis and ↓LDH	↑collagen density and ↑elastic fiber integrity	↑Nrf2 nuclear, ↑HO-1, and ↑NQO1 (Nrf2/ARE)	*In vitro*: HaCaT (UVB, 1–100 μg/mL, 24 h); *in vivo*: male ICR mice (UVA + UVB, topical, 0.67/1.33 mg/g, n = 10); clinical: none	III
3	Xingyu Chen, 2026, 10.2147/CCID.S587240	Danggui Buxue decoction (water decoction)	Not reported	↓ROS, ↓MDA, ↓SOD, ↑GSH-Px, and ↑SOD2	Not detected	↓P21 and ↓PPARα	↑MMP1	↑SOD2, ↑MMP1; ↓PPARα, and ↓P21	*In vitro*: HaCaT (H_2_O_2_, 400 μg/mL, 24 h); *in vivo*: none; clinical: none	IV
4	Hui Ke, 2024, 10.1016/j.jep.2024.118421	Sijunzi Tang (95% ethanol + ethyl acetate fraction)	Ginsenoside Rg5/Rh2, glycyrrhizin, liquiritin, polyporenic acid C, atractylenolide II (UPLC–Q-TOF–MS/MS)	↓ROS, ↓MDA, ↑T-SOD, ↑CAT, and ↑GSH-Px	Not detected	↓TNF-α, ↓IL-6, ↓IL-1β, ↓p16, ↓p53, ↓p21, and ↑mitochondrial membrane potential	↑collagen density and ↓epidermal thickening	↓p-p38, ↓p53, ↓p-p53, and ↓p21 (p38/p53/p21)	*In vitro*: rat EpiSCs (UVB, 15.6–62.5 μg/mL, 24 h); *in vivo*: male C57BL/6J mice (UVB, topical, 0.5%/2.0%, n = 6); clinical: none	III
5	Heng Li, 2022, 10.1016/j.asjsur.2022.01.043	Qibao Meiran Dan	Not reported	Not detected	Not detected	↓SA-β-gal, ↓apoptosis, ↓Bax, ↑Bcl-2, ↑LC3-II, and ↑proliferation	Not detected	↑Bcl-2, ↓Bax, and ↑LC3-II (apoptosis and autophagy)	*In vitro*: NHSFs (H_2_O_2_, single concentration, 24 h); *in vivo*: none; clinical: none	IV
6	Dan Ke, 2022, 10.1111/jocd.14908	Qibao Meiran Dan (drug-containing serum)	Not reported (72 compounds predicted using network pharmacology)	Not detected	Not detected	↓SA-β-gal and ↓TNF-α	↓MMP-1	↓TNF-α and ↓MMP-1 (TNF signaling)	*In vitro*: NHSFs (H_2_O_2_, 10% serum, 24 h); *in vivo*: female SD rats (for serum preparation, intragastric, 3.1 g/kg, 7days, n = 10); clinical: none	IV
7	Li-Ming Tian, 2020, 10.1016/j.jtv.2020.06.001	Yang Yan Qing E Wan (drug-containing serum)	Not reported	↓ROS, ↑SOD	Not detected	↓SA-β-gal, ↓p16INK4a	Not detected	↑β-catenin, ↓p16INK4a (β-catenin/p16)	*In vitro*: NHSFs (H_2_O_2_, 10% serum, 24 h); *In vivo*: female SD rats (for serum preparation, intragastric, 6.173 g/kg, n = 10); Clinical: none	IV
8	Shan Zhu, 2024, 10.1016/j.jep.2023.116996	Anti-aging formula (water/ethanol)	113 compounds including catalpol, ginsenosides, and flavonoids (UHPLC–Q-TOF–MS/MS)	Not detected	↓DNA damage (comet assay)	↓SA-β-gal, ↓SAHF, ↓p16, ↓p21, and ↓G0/G1 arrest	↑collagen/elastic fibers, and ↓epidermal thickening	↓CXCR2, ↓p-P38, and ↓P53 (CXCR2/P38/P53)	*In vitro*: HSF (AAPH, 100–500 μg/mL, 24 h); *in vivo*: ICR mice (UV, intragastric, 190.89/381.78 mg/kg, n = 8); clinical: none	III
9	Yuanfang Sun, 2026, 10.3389/fragi.2026.1768671	Anshen Bunao syrup (water extraction)	159 compounds including icariin, baohuoside I, and glycyrrhizin (UPLC–Q-TOF–MS)	↑SOD, ↑GSH-Px, and ↓MDA	Not detected	↓IL-1β, ↓IL-6, ↓TNF-α, ↓CRP, ↑IL-10, and ↓TGF-β	↓MMP-1, ↓epidermal thickness, and ↑elastic fiber	↓CYP2J10, ↓CBR3, ↑LPAR3 (sphingolipid and arachidonic acid metabolism)	*In vitro*: none; *in vivo*: male SD rats (D-gal, intragastric, 1.05–4.20 mL/kg/d, n = 6–10); clinical: none	III
10	Miao Han, 2022, 10.2147/CCID.S344138	Bazhen Tang (drug-containing serum)	Kaempferol, quercetin, β-sitosterol, stigmasterol, and naringenin (TCMSP prediction)	Not detected	Not detected	↓SA-β-gal and ↓p16INK4a	Not detected	↓p16INK4a; inhibits MAPK, TNF, and AGE–RAGE	*In vitro*: HaCaT (UVB, 10% serum, 24 h pre + 5 days irradiation); *in vivo*: male SD rats (for serum preparation, intragastric, 2.5 g/kg, n = 10); clinical: none	IV
11	Qinqian Tao, 2025, 10.2147/CCID.S552093	Five-Colored plant combination	Not reported (27 compounds predicted using network pharmacology)	Not detected	Not detected	↓SA-β-gal	↑collagen fibers, ↑Col IV, and ↑epidermal thickness	Regulates AGE-RAGE, PI3K–Akt, p53, and estrogen pathways; targets CBS, CDK5, and AKR1B10	*In vitro*: 3T3 (H_2_O_2_, 0.5%, 24 h); ex vivo: human isolated skin (UVA/UVB); *in vivo*: none; clinical: none	IV
12	Jinfan Xu, 2023, 10.1111/jocd.15542	MP Gel (mixed extracts + probiotic fermentation)	β-sitosterol, stigmasterol, oleanolic acid, shikonin, and acetylshikonin (TCMSP prediction)	↑SOD and ↓MDA	Not detected	↓Caspase-3	↑collagen arrangement and ↓epidermal thickness	↓Caspase-3; Predicted TNF, TP53, IL-17, and AGE–RAGE pathways	*In vitro*: none; *in vivo*: female KM mice (UV, topical, n = 10); clinical: none	III
13	Yingying Lin, 2025, 10.1111/jocd.70124	Wan’an Wan (30% 1,3-butanediol ultrasonic)	Morroniside, loganin, schisandrin A, pinoresinol diglucoside, and β-ecdysterone (HPLC)	↓NO, ↓tyrosinase	Not detected	Rescued MGO-impaired proliferation/adhesion/migration	↑COL1, ↑FN1, ↑LM5, ↑TNC, ↓MMP-2, and ↓MMP-9	↑TGF-β1, ↑filaggrin, ↑AQP3, ↑HAS2; ↓MMP-2/9; Predicted MAPK, and PI3K–Akt pathways	*In vitro*: HFF-1 (MGO, 0.5%–2%, 48 h); *in vivo*: none; clinical: none	IV
14	Chunbo Feng, 2024, 10.1016/j.heliyon.2024.e26131	Five-herb combination	Ginsenosides Rg1/Rb1/Re, kaempferol, liriodendrin, quercetin-3-O-β-D-galactoside, caffeic acid (HPLC–MS/NMR)	Not detected	↓γH2AX	↓SA-β-gal	↑COL1A1 and ↑COL3A1	Predicted FoxO, mTOR, longevity, and AGE–RAGE pathways	*In vitro*: NHDF (H_2_O_2_, 0.5%, 2 h); *in vivo*: none; clinical: none	IV
15	Yuning Jiang, 2025, 10.1016/j.phymed.2025.157360	Bazi Bushen capsule (water/ethanol)	Verbascoside, epimedin A, isoquercitrin, hyperoside, icariin, baohuoside I, chlorogenic acids, catalpol (UPLC–MS/MS)	↓ROS, ↓MDA, ↑T-SOD, ↑GSH-Px, and ↑NRF2 activation	↓8-OHdG	↓SA-β-gal, ↓p16, ↓TNF-α, ↓IL-1β, ↓IL-6, and ↓NF-κB	↑collagen/elastic fibers, ↓MMP1, and ↑hydroxyproline	Verbascoside/epimedin A bind KEAP1 → ↑NRF2 nuclear → ↑HO-1, ↑NQO1, and ↑SOD1; inhibits NF-κB	*In vitro*: RDF, HFSCs (UVA, 50/100 μg/mL BZBS or monomers); *in vivo*: male Lgr5-EGFP mice (UVA + UVB, intragastric, 1/2 g/kg/d, n = 10); clinical: none	III
16	Zhe Xu, 2023, 10.1111/jcmm.17833	Bazi Bushen capsule (water/ethanol)	Not detected	↑SOD, ↑GSH-Px, ↑T-AOC, and ↓MDA	Not detected	↓SA-β-gal, ↓p16, ↓p21, ↓p53, ↓IL-6, ↓IL-1β, ↓TNF-α, ↓Bax, ↓Cleaved caspase-3, ↑Bcl-2, ↑LC3-II/LC3-I, and ↓p62	↑hydroxyproline, ↑dermal thickness, ↓MMP-2, ↓MMP-9, and ↑COL17A1	Not detected	*In vitro*: none; *in vivo*: male SAMP6 mice (spontaneous aging, intragastric, 2.8 g/kg/d, n = 10); clinical: none	III
17	Mo Zhao, 2025, 10.1111/jocd.70280	Bazi Bushen capsule	Quercetin, kaempferol, arachidonate, suchilactone, ammidin, deoxyharringtonine, sitosterol, mandenol, ethyl linolenate, stigmasterol, poriferast-5-en-3beta-ol, and cholesterol (TCMSP/TCMBANK/SuperTCM)	Not detected	Not detected	Not detected	Not detected	Predicted MAPK, PI3K-Akt, and JAK–STAT signaling pathways	*In vitro*: none; *in vivo*: none; clinical: female volunteers with skin laxity (prospective single-group trial, 12-week oral administration, 0.4g/capsule, 2 capsules three times daily, n = 35)	II

Evidence level definition (adapted for this review):

• I–High-quality randomized controlled trial (RCT, double-blind, placebo-controlled, adequate sample size, predefined endpoints).

• II–Clinical trial with incomplete reporting (e.g., missing registration number, primary endpoint, or randomization details), non-randomized clinical trial, or single-arm clinical study (human data without placebo control or blinding).

• III–*In vivo* animal study (with or without *in vitro* cell data; includes efficacy endpoints in animal models of skin aging).

• IV–*In vitro* cell study only (molecular mechanism studies without animal or clinical validation; includes *ex vivo* human skin models without live animals).

Abbreviations: ↑/↓ indicate increase or decrease of the parameter; “Not detected” means that the study did not report or examine that phenotype.

### 4.1 Antioxidant effects and Nrf2/ARE pathway regulation

The Nrf2 pathway is the most frequently validated antioxidant mechanism in TCM formula research. Of the studies in this category, the highest evidence level is level III. No human data (level II or higher) support Nrf2 activation by any TCM formula for skin aging. Among the four formula studies reporting Nrf2 activation, only the modified Qing’e formula used siRNA knockdown to test causality; the remaining studies rely on nuclear translocation or downstream marker changes; whether Nrf2 is a necessary mediator or a non-specific stress response remains unclear.

The Erzhi formula (Liu et al., 2023) (level III) utilized UPLC to identify seven compounds, including salidroside, specnuezhenide, and isoquercitrin. In UVB-induced HaCaT cells and a UVA/UVB-induced mouse photoaging model, it was demonstrated to reduce ROS, MDA, and LDH levels; increase SOD, CAT, and GSH-Px activity; decrease DNA damage and apoptosis; and activate HO-1 and NQO1 by promoting Nrf2 nuclear translocation. However, this study validated only downstream effectors of the Nrf2 pathway, did not explore upstream regulatory molecules, and did not investigate the compatibility proportion or multi-pathway mechanisms of *Ligustrum lucidum* Aiton (Oleaceae) and *Eclipta prostrata* Lour. (Asteraceae).

A modified Qing’e formula (Zhu et al., 2022) (level III) was used to tentatively identify 19 bioactive compounds using UHPLC–Q-TOF–MS, although only qualitatively without quantitative determination. In HaCaT cells and ICR mouse photoaging models, it was shown to increase cell viability, reduce ROS and MDA levels, increase SOD/CAT/GSH-Px activity, and repair collagen and elastic fibers. Mechanistically, it promoted Nrf2 nuclear translocation and activated HO-1 and NQO1, with Nrf2 siRNA knockdown reversing these protective effects. This study is one of the few among the reviewed reports that conducted Nrf2 siRNA knockdown reverse validation, significantly strengthening the causal evidence for its mechanism compared with that of similar studies. The absence of human clinical data means that even stronger evidence could be obtained from future trials.

Danggui Buxue decoction (Chen et al., 2026) (level IV), as demonstrated in H_2_O_2_-induced HaCaT cells, scavenges ROS, reduces the activity of SOD/MDA, and exerts protective effects by upregulating the expression of SOD2 and MMP1 while downregulating the expression of PPARα and P21. The increase in MMP1 requires interpretation: under acute H_2_O_2_ injury, cells temporarily downregulate MMP1 to preserve matrix integrity; the decoction-mediated restoration of MMP1 may represent normalization of collagen metabolism. Unfortunately, this study is of very low evidence level—it was confined to *in vitro* experiments, did not identify bioactive compounds, and did not explore the contribution of the compatibility ratio between *Astragalus membranaceus* (Fisch.) Bunge (Fabaceae) and *Angelica sinensis* (Oliv.) Diels (Apiaceae).

Sijunzi Tang (Ke et al., 2024) (level III) integrated network pharmacology, molecular docking, and UPLC–QTOF–MS/MS to screen core compounds, such as ginsenosides Rg5/Rh2, glycyrrhizin, liquiritin, polyporenic acid C, and atractylenolide II, predicting their targeting of TP53, CDKN2A, TNF, and IL6 to regulate the p38/p53/p21 pathway. *In vivo* and *in vitro* experiments confirmed its ability to ameliorate UVB-induced skin photoaging in mice, increase the activity of antioxidant enzymes, elevate collagen fiber content, reduce SASP factors, and maintain epidermal stem cell stemness. This study links the antiaging effects of Sijunzi Tang to the ethyl acetate fraction; however, the contribution of water-soluble compounds was not clarified, and the study lacked transdermal absorption and human clinical data, resulting in insufficient translational evidence.

### 4.2 Regulation of cellular senescence, apoptosis, and autophagy

All studies in this category are classified as level III or level IV. No human data are available. Across the seven studies in this section, only the anti-aging formula performed gene silencing/overexpression rescue experiments (CXCR2); the others rely on SA-β-gal and p16/p21 as readouts without evaluating upstream mechanisms or skin specificity.

Two studies on Qibao Meiran Dan focused on the apoptosis‒autophagy balance and cellular senescence. The first study (Li H. et al., 2022) (level IV) demonstrated that in an H_2_O_2_-induced fibroblast senescence model, Qibao Meiran Dan increased cell viability, reduced SA-β-gal positivity and apoptosis rates, upregulated Bcl-2, downregulated Bax, and increased LC3II positivity and expression levels, suggesting that it plays a role in delaying cellular senescence by regulating the apoptosis‒autophagy balance. The second study (Ke et al., 2022) (level IV) used network pharmacology to screen 72 bioactive compounds and 273 targets, identifying 64 core targets related to skin aging that were enriched in the TNF signaling pathway. *In vitro* experiments confirmed that Qibao Meiran Dan-containing serum reduced H_2_O_2_-induced SA-β-gal positivity in fibroblasts and downregulated TNF-α and MMP-1 expression. Both studies lacked identification and quantification of the formula’s bioactive compounds and measured only a limited set of indicators, resulting in insufficient mechanistic depth that fundamentally undermines the reliability of their conclusions.

Yang Yan Qing E Wan (YYQEW) (Tian et al., 2020) (level IV) attenuated H_2_O_2_-induced premature senescence in human skin fibroblasts by reducing SA-β-gal activity and ROS production, increasing SOD activity, upregulating β-catenin expression, and downregulating p16^INK4a^ expression. These findings suggest that YYQEW exerts anti-skin aging effects *via* regulating the β-catenin/p16^INK4a^ signaling pathway. This study shares the same limitations as the Qibao Meiran Dan studies: it lacks bioactive compound identification and quantification, measures only a limited set of indicators, and uses an *in vivo* protocol solely to obtain drug-containing serum for subsequent *in vitro* experiments. Therefore, its evidence level was downgraded to level IV.

The anti-aging formula (Zhu et al., 2024) (level III) has been confirmed in UV-induced skin photoaging mouse models and AAPH-induced human skin fibroblast senescence models to increase skin moisture content, reduce wrinkles and pigmentation, increase collagen and elastic fiber content, and downregulate P21 and P16. Further transcriptome sequencing identified CXCR2 as a core target, and CXCR2 silencing and overexpression experiments verified that the anti-aging formula exerts anti-senescence effects by inhibiting the CXCR2/P38/P53 pathway. This study links a TCM formula with the CXCR2-mediated senescence signaling axis, incorporating gene silencing and overexpression rescue assays, providing a mechanistic depth that exceeds that of most similar studies.

Anshen Bunao Syrup (Sun et al., 2026) (level III) was analyzed using UPLC‒Q-TOF‒MS, and 159 chemical compounds were characterized. In D-galactose-induced aging rats, it improved motor and cognitive function while alleviating skin aging and neuronal damage. Integrated transcriptomic and metabolomic analyses revealed the regulation of CYP2J10, CBR3, LPAR3, as well as sphingolipid and arachidonic acid metabolic pathways. This study only assessed skin *via* HE staining (epidermal thickness/elastic fiber morphology) without collagen quantification or elastin-specific assays, leading to insufficient evidence for its skin anti-aging efficacy.

Bazhen Tang (Han et al., 2022) (level IV) predicted 160 bioactive compounds and identified 60 core targets related to photoaging through network pharmacology. These targets were enriched in the MAPK, TNF, and AGE–RAGE signaling pathways. Molecular docking confirmed the strong binding of kaempferol, quercetin, β-sitosterol, and naringenin with PTGS2, CASP3, MAPK1, and MAPK3. *In vitro* experiments revealed that drug-containing serum reduced the rate of UVB-induced SA-β-gal positivity and p16^INK4a^ expression in HaCaT cells. The major limitation of this study is that the bioactive compounds in the drug-containing serum remain unidentified, and the overall experimental evidence is too weak.

The five-colored plant combination (Tao et al., 2025) (level IV)—a formula based on the Five Elements and Five Colors theory of TCM, consisting of mung bean, white truffle, black truffle, *Chrysanthemum × morifolium* (Ramat.) Hemsl. (Asteraceae), and *Lycium barbarum* L. (Solanaceae)—was established. Network pharmacology identified 27 core compounds and 636 targets, with key targets CBS, CDK5, and AKR1B10 and enriched pathways including AGE-RAGE, PI3K-Akt, p53, and estrogen signaling. Subsequent fibroblast scratch assays, senescence β-galactosidase staining, and *ex vivo* human skin models confirmed that at 0.5% concentration, it significantly accelerated fibroblast migration, reduced senescent cells, and increased epidermal viable cell layer thickness, collagen fiber content, and type IV collagen. Although the formula incorporates an innovative TCM theoretical framework, the experimental evidence is very limited, and the predicted mechanisms have not been experimentally validated.

MP gel (Xu J. et al., 2023) (level III) contains *Cnidii monnieri* (L.) Cusson (Apiaceae), Arnebiae *euchroma* (Royle ex Benth.) I.M.Johnst. (Boraginaceae), *A. sinensis* (Oliv.) Diels (Apiaceae), Poria cocos (Schw.) Wolf (Polyporaceae), Borneolum, and probiotic fermentation supernatant. Network pharmacology identified core compounds including β-sitosterol and stigmasterol, which target TNF, TP53, CASP3, IL6, MMP9, and other key nodes. In a UV-induced mouse photoaging model, MP gel reduced wrinkles, lowered MDA levels, increased SOD activity, and downregulated Caspase-3 expression at the mRNA and protein levels. This study integrates traditional formulae, probiotic fermentation, and network pharmacology but lacks quantification of bioactive compounds, cellular-level validation, and analysis of the multi-pathway mechanisms between the formula and probiotics.

### 4.3 Regulation of ECM metabolism

Of the three ECM-related formula studies, none measured upstream signaling pathways in a validated manner; most reported only collagen or MMP changes without mechanistic depth, similar to the pattern observed in single-botany studies.

Wan’an Wan (WAW) formula (Lin et al., 2025) (level IV) used network pharmacology to identify 412 skin-related targets and enriched pathways, including MAPK and PI3K/Akt. HPLC confirmed key compounds, such as morroniside, loganin, and schisandrin A. Cell experiments verified that the optimized formula inhibits NO production and tyrosinase activity, upregulates filaggrin, AQP3, HAS2, and TGF-β1, downregulates MMP-2/9, and rescues MGO-induced fibroblast dysfunction (proliferation, adhesion, and migration). Unlike most skin aging studies that rely on UV or D-galactose models, this study employed a methylglyoxal (MGO)-induced HFF-1 cell model, which specifically captures glycation-driven endogenous aging (Nowotny et al., 2018; Liu et al., 2022). Moreover, the outcome measures focused on skin barrier function (filaggrin and AQP3) and ECM remodeling rather than conventional oxidative stress markers, providing a distinct mechanistic perspective.

The five-herb combination (Feng et al., 2024) (level IV) was compared with single *P. ginseng* C.A.Mey. (Araliaceae) (one-herb) and the three-herb combination [*P. ginseng* C.A.Mey. (Araliaceae) + *Polygonatum cyrtonema* Hua (Asparagaceae) + *Epiphyllum oxypetalum* (DC.) Haw. (Cactaceae)]. The five-herb formulation [*P. ginseng* C.A.Mey. (Araliaceae)+ *P. cyrtonema* Hua (Asparagaceae) + *E. oxypetalum* (DC.) Haw. (Cactaceae)+ *Nelumbo nucifera* Gaertn. (Nelumbonaceae) + *Osmanthus fragrans* Lour. (Oleaceae)] significantly upregulated COL1A1/COL3A1 mRNA (type I/III collagen synthesis), reduced H_2_O_2_-induced SA-β-gal-positive senescent cells, and decreased γH2AX DNA damage, showing stronger multi-pathway anti-aging effects. However, the study lacks mechanistic depth and remains largely superficial—no signaling pathways were investigated, and only phenotypic endpoints were reported.

### 4.4 Typical research case

The Bazi Bushen capsule is the most systematically studied and relatively well-evidenced formula in this group of studies. These three studies explored antioxidant effects, multidimensional homeostasis regulation, and clinical efficacy, respectively. Notably, none of these studies reached level I; all were classified as level II or level III. As observed in the single-botany section, even the best-studied formula lacks gene knockout or rescue validation *in vivo*, and its clinical component provides no molecular data.

At the level of antioxidant mechanisms, the Bazi Bushen capsule (Jiang et al., 2025) (level III) was demonstrated in a UVA + UVB-induced mouse photoaging model to alleviate wrinkles, restore collagen and elastic fiber structure, increase SOD/GSH-Px activity, and reduce MDA and ROS. Using network pharmacology, molecular docking, and bio-layer interferometry (BLI), verbascoside and epimedin A were confirmed to directly bind KEAP1, thereby promoting NRF2 nuclear translocation and activating downstream HO-1/NQO1/SOD1 antioxidant pathways.

At the level of cellular senescence and multidimensional homeostasis regulation, the Bazi Bushen capsule (Xu Z. et al., 2023) (level III) demonstrated regulatory effects in the SAMP6-accelerated aging mouse model: after 9 weeks of intervention, it increased dermal thickness and hydroxyproline content, elevated SOD/GSH-Px/T-AOC activities, reduced MDA and IL-6/IL-1β/TNF-α/MMP2/MMP9 levels, decreased SA-β-gal positivity and p16/p21/p53 expression, inhibited Bax/cleaved caspase-3 while increasing Bcl-2, elevated the LC3-II/LC3-I ratio, reduced p62 expression, and upregulated OCT4/SOX2/NANOG and COL17A1/ITGA6. This study validated the anti-skin-aging effects of Bazi Bushen from five dimensions: oxidative stress, inflammation/SASP, apoptosis, autophagy, and epidermal stem cell homeostasis, representing one of the most phenotypic studies in the reviewed literature. However, notable limitations include the following: only a single dose was assessed, no specific signaling pathway validation was performed, and clinical data were lacking, resulting in thorough phenotypic characterization but insufficient mechanistic causal evidence.

At the level of clinical translation, the Bazi Bushen capsule (Zhao et al., 2025) (level II) provides human clinical evidence for skin anti-aging. After 12 weeks of intervention in 35 female volunteers, the average wrinkle depth was significantly reduced, and skin elasticity and moisture content were significantly improved. This is the only clinical study in the reviewed literature that validates TCM formula efficacy for skin laxity, providing preliminary clinical evidence for TCM anti-skin-aging effects. The study has several limitations, including a single-arm design without placebo or active control and a 12-week observation period without long-term safety data.

Overall, research on the Bazi Bushen capsule still holds landmark significance in the field of compound anti-skin aging—it has constructed a relatively complete evidence chain from molecular targets (KEAP1–NRF2) and multidimensional phenotypes (SAMP6 model) to clinical exploration (single-arm trial).

### 4.5 Common methodological limitations and overall evaluation of evidence levels

Based on 17 studies, the following high-frequency common deficiencies can be summarized: (1) network pharmacology “emphasizes prediction over validation”—more than half of the studies rely on network pharmacology to screen targets, but only a few studies have used genetic validation, while the remaining studies remain at the level of molecular docking or protein expression detection for correlation; (2) severe lack of compound quality control—only a few studies, such as those on the Erzhi formula, Anshen Bunao syrup, modified Qing’e formula, and Sijunzi decoction, have identified or quantified bioactive compounds. Most compound formulae did not establish a chromatographic fingerprint or content determination standards, making batch-to-batch consistency unverifiable; (3) absence of formula decomposition and compatibility mechanisms—except for the five-herb formula, which compared differences between single botanical drugs and formulae, other studies failed to investigate the contributions of “monarch, minister, assistant, and guide” botanical drugs or determine optimal compatibility ratios, leaving the molecular basis of multi-pathway effects unclear; (4) gender bias and model uniformity—among the 11 studies that explicitly reported animal sex, seven used only male subjects, three used only female subjects, and one did not report. Most employed a single aging model (D-galactose, UV, or SAMP6), lacking cross-validation across multiple models; (5) clinical translation gap—only the Bazi Bushen capsule provided a single-arm clinical trial, with no randomized double-blind placebo-controlled trials. No studies reported transdermal absorption rates, systemic exposure, or long-term safety data for the compound formulas; (6) incomplete ethical and sourcing documentation for animal-derived materials—among the 17 formula studies, two preparations—Anshen Bunao syrup and Bazi Bushen capsule—include animal-derived components [*Cervus nippon* Temminck (Cervidae) antler and, in the latter, *Hippocampus sp*. (Syngnathidae)]. However, the original articles did not report the origin, farming conditions, or sustainable harvesting practices for these materials. Although their inclusion does not invalidate the preclinical findings, this information gap prevents a full assessment of batch-to-batch consistency and compliance with current ethnopharmacological best-practice standards regarding animal welfare. Overall, similar to the single-botany section, the formula evidence base is predominantly preclinical, and the few genetic validation attempts remain *in vitro* only. Most studies rely on correlative markers without proving the necessity or sufficiency of the claimed targets.

Across the 36 studies reviewed, Tables 3 and 4 summarize representative bioactive compounds with reported target engagement data and the quantitative reporting frequency of each major pathway, respectively. Nrf2/ARE activation is the most consistently reported mechanism (21/36 studies) and the only one with causal validation, whereas MAPK/AP-1 inhibition and MMP-mediated ECM protection are secondary, and PI3K/Akt/mTOR, NAD^+^/Sirtuin, and TGF-β/Smad are less frequent and context-dependent. Notably, no study has demonstrated pharmacological synergy beyond additive effects, suggesting that most “multi-pathway” claims reflect parallel activation rather than emergent interactions.

**TABLE 3:** Representative bioactive compounds and their target engagement evidence.

Compound	Chemical structure	Molecular formula	Source	Primary target/mechanism	Docking/binding evidence	References
4,5-Dicaffeoylquinic acid	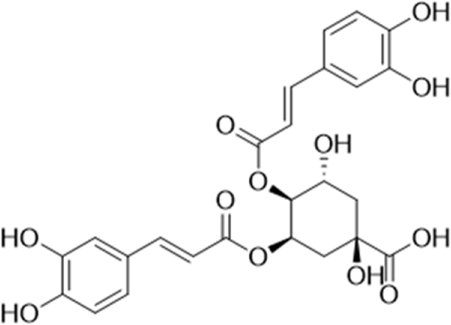	C_25_H_24_O_12_	*Chrysanthemum × morifolium* (Ramat.) Hemsl. (Asteraceae) (single)	↑Nrf2 nuclear translocation	Functional validation at extract level (Western blot: ↑Nrf2 nuclear translocation, ↑HO-1/NQO1 in HaCaT and HFF-1); no docking or binding data for purified compound	Xu et al. (2024a)
Baicalin	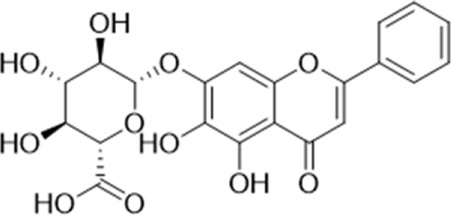	C_21_H_18_O_11_	*Scutellaria baicalensis* Georgi (Lamiaceae) (single)	REV-ERBα antagonist → BMAL1 ↑	Functional validation at extract level (Gal4 chimeric, Bmal1-Luc reporter, and qPCR/WB); no direct binding data for purified baicalin	Sun et al. (2022)
Epimedin A	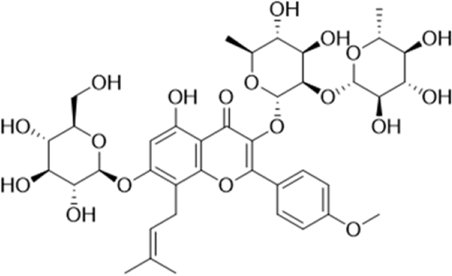	C_39_H_50_O_20_	Bazi Bushen (formula)	KEAP1-NRF2 binding	Experimentally validated direct binding (BLI: KD = 71.90 μM; molecular docking: −10 kJ/mol; ARE-luc, NRF2 nuclear translocation, and siRNA/ML385 rescue)	Jiang et al. (2025)
Ginsenoside Rg5	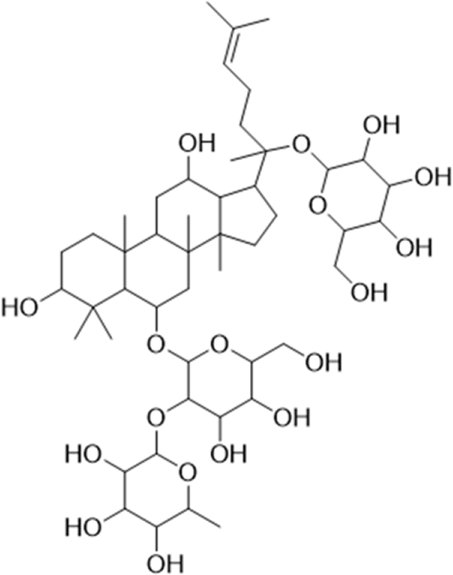	C_42_H_70_O_12_	Sijunzi Tang (formula)	↓p38/p53/p21 pathway	Predicted docking (AutoDock Vina: TP53; not experimentally validated)	Ke et al. (2024)
Kaempferol	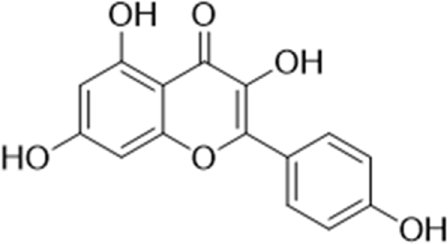	C_15_H_10_O_6_	Bazhen Tang (formula)/*Hibiscus mutabilis*. L. (Malvaceae) (single)	↓AKT/STAT3 pathway	Predicted docking (AutoDock: PTGS2/MAPK1/CASP3/TP53 and AKT1/TNF-α/STAT3/MMP9/EGFR; not experimentally validated)	Han et al. (2022), Chen et al. (2024)
Luteolin	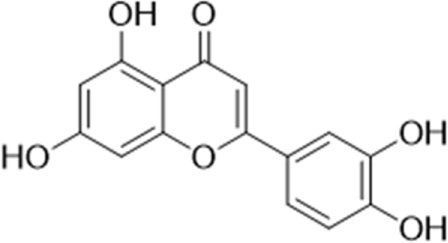	C_15_H_10_O_6_	*Cardiospermum halicacabum* L. (Sapindaceae) (single)	↓PI3K/Akt/mTOR	Functional validation at extract level (RNA-seq and Western blot: ↓PI3K/Akt/mTOR); no docking or binding data for purified luteolin	Zhao et al. (2026)
Paeonol	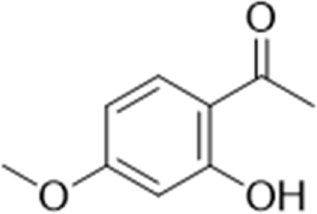	C_9_H_10_O_3_	*Paeonia suffruticosa* Andr. (Paeoniaceae) (single)	↑Nrf2/ARE, ↓MAPK/AP-1	Functional validation with purified paeonol (Sun et al., 2018) and extract-level validation (Liu et al., 2025); no docking or binding data in either study	Sun et al. (2018); Liu et al. (2025)
Quercetin	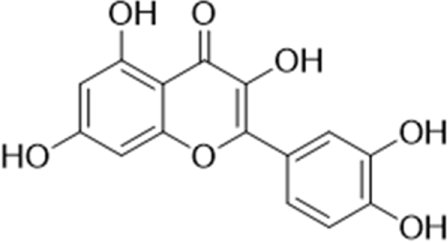	C_15_H_10_O_7_	Bazhen Tang (formula)/*Hibiscus mutabilis*.L. (Malvaceae) (single)	↓AKT/STAT3 pathway	Predicted docking (AutoDock: PTGS2/MAPK1/CASP3/TP53 and AKT1/TNF-α/STAT3/MMP9/EGFR; not experimentally validated)	Han et al. (2022); Chen et al. (2024)
Rosmarinic acid	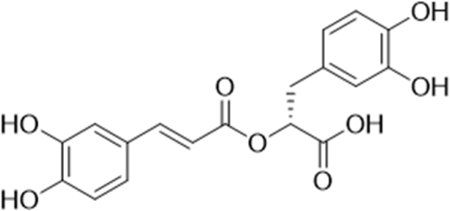	C_18_H_16_O_8_	*Prunella vulgaris* L. (Lamiaceae) (single)/Modified Qing’e Formula (formula)	TGF-β/Smad↑, Nrf2/ARE	Functional validation at extract level (PVE + MQEF; no docking or binding data for purified compound)	Zhang et al. (2018); Zhu et al. (2022)
Verbascoside	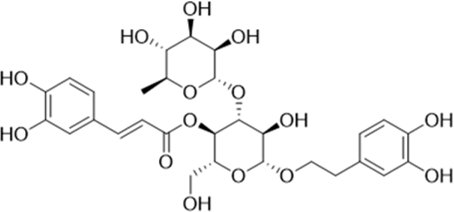	C_29_H_36_O_15_	Erzhi formula (formula)/Bazi Bushen (formula)	KEAP1–NRF2 binding	Experimentally validated direct binding (BLI: KD = 64.15 μM; molecular docking: −9.2 kcal/mol; ARE-luc, NRF2 nuclear translocation, and siRNA/ML385 rescue)	Liu et al. (2023); Jiang et al. (2025)

Source classification:

• Single—Compound derived from a single botanical drug/extract study.

• Formula—Compound identified in a polyherbal formula study.

Target engagement evidence classification:

• Experimentally validated direct binding—Direct molecular interaction confirmed by biophysical assays (BLI or SPR) with quantitative binding affinity (KD values), supplemented by cellular rescue experiments (siRNA knockdown or pharmacological inhibition).

• Functional validation at the extract level—Pathway modulation demonstrated in cell-based assays (e.g., Western blot, luciferase reporter, and qPCR) using the whole extract; however, direct binding data for the purified compound are not available, and causality at the compound–target level remains unconfirmed.

• Predicted docking—In silico molecular docking predictions only; no experimental validation (neither binding assays nor functional rescue) has been performed.

Abbreviations: ↑/↓ indicate increase or decrease of the parameter; BLI, bio-layer interferometry; KD, equilibrium dissociation constant; ARE, antioxidant response element; PVE, Prunella vulgaris L. (Lamiaceae) extract; MQEF, Modified Qing’e Formula.

**TABLE 4:** Cross-study summary of mechanistic pathways, reporting frequency, and causal validation status.

Pathway/Mechanism	Single-botanical drug studies (n = 19)	Formula studies (n = 17)	Total (N = 36)	Studies with causal validation (n)	Evidence strength
Nrf2/ARE activation	13	8	21	3 (siRNA, KEAP1-binding)	Relative evidence strength: highest among reviewed mechanisms
MMP inhibition/ECM protection	10	4	14	0	Moderate
MAPK/AP-1 inhibition	8	3	11	0	Moderate
PI3K/Akt/mTOR suppression	5	2	7	0	Low
NAD+/Sirtuin modulation	3	1	4	0	Low
TGF-β/Smad regulation	2	1	3	0	Low
CXCR2/p38/p53	0	1	1	1 (CXCR2 silencing)	Emerging

Evidence level definition (adapted for this review):

• “Relative evidence strength: highest among reviewed mechanisms”—The pathway with the most robust causal validation (three studies: siRNA knockdown or KEAP1 direct binding) and the highest reporting frequency (21/36 studies), indicating the strongest mechanistic support in the current literature.

• “Moderate”—Pathways reported in 11–14 studies but lacking causal validation (no genetic manipulation or direct binding assays); evidence remains correlative.

• “Low”—Pathways reported in fewer than 10 studies without causal validation; evidence is sparse and hypothesis-generating.

• “Emerging”—A pathway identified in only one study but with causal validation (CXCR2 silencing), suggesting potential mechanistic relevance that requires further confirmation in additional models.

Abbreviations: BLI, bio-layer interferometry; siRNA, small interfering RNA.

## 5 Limitations and future perspectives

As a narrative review, this synthesis is inherently selective. We did not perform a formal quality assessment or quantitative synthesis, and the literature search was limited to English-language articles indexed in PubMed and Web of Science. Thus, some relevant studies, particularly those published in Chinese journals, may have been omitted. Clinical evidence remains extremely limited and consists mainly of small human studies, including trials described as randomized or double-blind but incompletely reported, and single-arm evaluations; no adequately powered, preregistered, high-quality RCT has yet established clinical efficacy for TCM interventions in skin aging. Consequently, the molecular mechanisms discussed in this review are derived largely from preclinical findings, and this review makes no claim of clinical efficacy.

Another limitation is that all 36 studies included in this review reported exclusively positive outcomes. This pattern is unlikely to reflect the true state of evidence and strongly suggests publication bias, selective outcome reporting, or underpowered studies. A recent meta-analysis of 40 RCTs on dietary supplements for skin photoaging (Yang et al., 2025) offers an instructive contrast: although collagen, flavanols, and polyphenols showed significant benefits, other supplements—including hyaluronic acid, lycopene, and carotenoids—did not significantly improve skin elasticity or minimal erythema dose. Null or negative findings are rarely reported in the TCM literature, in contrast to the broader field of skin anti-aging research, a discrepancy that warrants explicit acknowledgment. To address these issues, existing reporting frameworks should be adopted. The CONSORT extension for herbal interventions (Gagnier et al., 2006) provides a checklist for reporting RCTs, including botanical identification, extraction, and compound quantification. The ConPhyMP statement (Heinrich et al., 2022) and ARRIVE 2.0 guidelines (Percie du Sert et al., 2020) offer standards for phytochemical characterization and animal studies, respectively. We therefore urge future TCM studies to report all findings regardless of directionality, to preregister study protocols, and to adhere to these established standards.

Future studies should address four priorities. First, appropriate *in vivo* or *in vitro* aging models should be selected according to the research question. For photoaging studies, standardized UV irradiation models [e.g., ICR mice as reported in previous studies (Liu et al., 2023; Chen et al., 2024; Xu S. et al., 2024)] are recommended; for intrinsic aging, naturally aged animals or senescence-accelerated mouse strains may be more suitable. Second, rigorous RCT designs—placebo-controlled, blinded, adequately powered—should be adopted, with prospective registration and adherence to CONSORT. Third, *in silico* docking and network pharmacology are indispensable front-end screening tools that predict bioactivity and prioritize compound–target pairs, guiding experimental design while reducing the cost and time burden of *in vivo* testing (Guo et al., 2022; Chihomvu et al., 2024). However, these predicted interactions remain hypothesis-generating and should be interpreted cautiously unless supported by experimental validation. Fourth, standardized quality control of TCM interventions is essential, including chromatographic fingerprinting and quantification of marker compounds. Furthermore, moving beyond conventional biochemical readouts by integrating multi-omics (transcriptomics, proteomics, and metabolomics) and, where feasible, pharmacodynamic‒pharmacokinetic network analysis can help distinguish bioavailable compounds from inert compounds (Wu et al., 2025; Wang et al., 2026). Such approaches may decode the physicochemical, pharmacokinetic, and pharmacodynamic basis of multi-component TCM formulas. International collaboration and cross-population validation are also needed to extend findings beyond East Asian cohorts.

## 6 Conclusion

This narrative review synthesized preclinical evidence from 19 single-botanical and 17 formula studies on TCM-derived interventions against skin aging. We focused on four core mechanisms: oxidative stress, cellular senescence, ECM degradation, and DNA damage. Across these studies, the most consistently reported effects include Nrf2/ARE activation, reduced ROS and MDA levels, increased SOD activity, MMP inhibition, and lower expression of senescence markers (p16, p21, and SA-β-Gal). These findings suggest multi-pathway modulation rather than true pharmacological synergy. However, clinical evidence remains extremely limited and consists mainly of small human studies, including trials described as randomized or double-blind but incompletely reported, and single-arm evaluations; no adequately powered, preregistered, high-quality RCT has yet established clinical efficacy for TCM interventions in skin aging. Thus, current findings are hypothesis-generating, not proof of clinical efficacy. Future research should prioritize well-designed RCTs and dose–response studies.
