# Supplementary material for: Molecular mechanisms of traditional Chinese medicine in skin aging: a narrative review
Source: Front Pharmacol. 2026 Aug 19;17:1792159. doi: 10.3389/fphar.2026.1792159 (PMC13536475; doi:10.3389/fphar.2026.1792159)
Supplement: Supplementary file 3 [file Table1.docx]

**Supplementary Materials**

**Supplementary Figure 1.** Flow diagram of the study selection process.

**Supplementary Table 1.** Pharmacological parameters of single botanical drug studies

| **Extract type** | **Analytical characterization** | **Model type** | **Induction method** | **Dose / Concentration range** | **Minimum effective concentration / dose** | **Treatment duration** | **Positive control** | **Negative control** | **Ref.** | **Four Pillars Compliance & Limitations** |
| --- | --- | --- | --- | --- | --- | --- | --- | --- | --- | --- |
| 80% ethanol extract of *Scutellaria baicalensis* Georgi (Lamiaceae) (SBG) | LC-MS/MS quantified wogonoside, baicalein, baicalin, wogonin | *In vivo*: C57BL/6 mice; *In vitro*: NIH3T3, HaCaT, mouse primary dermal fibroblasts, HEKa | *In vivo*: D-gal (250 mg/kg s.c. daily) + UVB (120 mJ/cm² daily) for 6 weeks; *In vitro*: D-gal 8 g/L (NIH3T3) or UVB 100 mJ/cm² (others) | *In vivo* (topical): 25, 100, 400 mg/kg; *In vitro*: 12.5, 25, 50, 100 μg/mL | *In vivo*: 25 mg/kg; *In vitro*: 12.5 μg/mL | *In vivo*: 4 weeks (daily after UVB); *In vitro*: 24 h post-induction | Vitamin C (40 mg/kg *in vivo*; *in vitro* concentration not reported) | Vehicle (saline for *in vivo*; medium for *in vitro*) | (Sun et al., 2022) | **Compliant** |
| 70% ethanol extract of *Cardiospermum halicacabum* L. (Sapindaceae) (CHE) | UPLC-Orbitrap-MS (31 compounds identified) + HPLC quantified myricitrin, luteolin, apigenin | Human skin fibroblasts (HSFs) | UVB irradiation (302 nm, 48 mJ/cm², 60 s at 15 cm) | 25, 50, 100 μg/mL (24 h pretreatment) | Not explicitly reported; lowest tested 25 μg/mL shows partial efficacy, optimal at 100 μg/mL | 24 h pretreatment + 24 h post-UVB culture | Vitamin E (VE) — concentration not reported | Untreated control & UVB-irradiated model group | (Zhao et al., 2026) | **Partially compliant** |
| Hot water extract of *Cordyceps cicadae* (Miq.) Massee (Cordycipitaceae) fruiting bodies (CCE) | HPAEC (polysaccharides: galactose:glucose:mannose = 6:22:13, MW: 2.217×10⁷, 3.752×10⁴, 2.316×10³ Da) & alditols (mannitol 107.00 mg/g, trehalose 65.25 mg/g, etc.); HPLC (8 nucleosides); Folin-Ciocalteu (polyphenols 3.8 mg/g); Bradford (protein 35.7 mg/g) | Human skin fibroblasts (from facial skin of 45-year-old woman) | No exogenous induction (normal fibroblasts; assessed HA synthesis promotion) | 5, 10, 50, 100, 500, 1000 μg/mL (viability); 50, 100 μg/mL (HA synthesis) | 5 μg/mL (viability); 50 μg/mL (HA synthesis) | 24 h (viability); 48 h (HA content); 24 h (RNA-seq) | Not reported (ascorbic acid used only for cell-free antioxidant assays) | Non-treatment (NT) group (regular DMEM, no CCE) | (Shao et al., 2023) | **Partially compliant** |
| 70% ethanol extract of *Paeonia suffruticosa* Andr. (Paeoniaceae) root (PSR); also tested pure paeonol (PA) | HPLC (paeonol detected as the only main compound at 179.88 μg/mL) | *In vitro*: HaCaT cells; *In vivo*: HR-1 hairless mice (female, 7-week-old) | *In vitro*: UVB 125 mJ/cm²; *In vivo*: chronic UVB (protocol cited from Hwang et al., 2014) | *In vitro* (PSR & PA): 1, 10 μg/mL; *In vivo* (topical): PA 0.1%, 1%; PSR 1%, 5% | *In vitro*: 10 μg/mL; *In vivo*: PA 0.1%, PSR 1% | *In vitro*: 24 h (MMP-1/ROS), 3 h (Nrf2), 30 min–4 h (MAPK/AP-1); *In vivo*: not explicitly stated | *In vitro*: Gallic acid (1, 10 μg/mL); *In vivo*: Retinyl palmitate (1%) | Untreated control & UVB-irradiated control | (Sun et al., 2018) | **Compliant** |
| AB-8 macroporous resin-purified extract of *Paeonia suffruticosa* Andr. (Paeoniaceae) root bark (PSAE); main components: PGG (10.79%) and paeonol (23.85%) | HPLC (PGG and paeonol quantified); total phenolic content 48.9% (Folin-Ciocalteu); HRMS for qualitative analysis | *In vitro*: HaCaT (UVB), HFF (UVA); 3D T-Skin™ (UVA); Human: forearm skin (aged 30–50) | HaCaT: UVB 0.2 mW/cm², 12 mJ/cm²; HFF: UVA 2 mW/cm², 15 J/cm²; 3D skin: UVA (parameters not specified) | HaCaT/HFF: 20, 40 μg/mL; 3D skin: 1% PSAE; Human: 2.0 ± 0.1 mg/cm² | *In vitro*: 20 μg/mL (both HaCaT and HFF) | *In vitro*: 2 h pretreatment + 1 h post-UV (ROS); 24 h (gene/protein); 3D skin: 24–48 h; Human: single application, assessed at 2, 4, 6 h | Antioxidant assays: Vitamin C, Tetracycline, Dipotassium Glycyrrhizinate; Cell/3D models: not reported | Blank control (no UV, no PSAE); Model/UV-irradiated control; Vehicle control (human) | (Liu et al., 2025) | **Partially compliant** |
| Water extract of *Chrysanthemum × morifolium (Ramat.) Hemsl. (Asteraceae)* buds, purified by D101 macroporous resin (50% ethanol fraction) (CE) | HPLC-Q-TOF-MS/MS (16 polyphenols identified); HPLC quantified 7 components: 4,5-dicaffeoylquinic acid (31.90%), 3,4-dicaffeoylquinic acid (10.84%), luteolin-7-O-glucoside (9.03%), apigenin-7-O-glucoside (8.56%), acacetin-7-O-galactoside (6.53%), 3,5-dicaffeoylquinic acid (6.05%), diosmetin-7-O-glucoside (4.43%) | *In vitro*: HaCaT, HFF-1; *In vivo*: ICR mice (female, 4–5 weeks old) | *In vitro*: UVB 240 mJ/cm²; *In vivo*: UVB 40 mJ/cm² per session, every 2 days after first week, total 4 weeks | *In vitro*: 50, 75, 100 μg/mL; *In vivo* (topical): CE low 0.208 mg/cm²/day, CE high 0.417 mg/cm²/day | *In vitro*: 50 μg/mL; *In vivo*: 0.208 mg/cm²/day | *In vitro*: 24 h (viability), 6 h (ROS), 8 h (WB), 24 h (GSH); *In vivo*: 4 weeks | Antioxidant assays: Ascorbic acid; *In vivo*: Ascorbic acid (0.167 mg/cm²/day); *In vitro* cell models: not reported | Group C (no UVB, no CE); Group U (UVB only); Group UN (UVB + solvent vehicle) | (Xu et al., 2024) | **Partially compliant** |
| 50% ethanol extract of rice bran (L. (Poaceae)), fermented with *Aspergillus oryzae* (Ahlb.) Cohn (Trichocomaceae) for 14 days (RBE) | UPLC-QTOF-MS/MS (8 compounds identified: azelaic acid, ferulic acid, pinellic acid, 9,10,13-trihydroxy-11-octadecenoic acid, γ-tocotrienols, δ-tocotrienols, dihydroferulic acid dimer, squalene) | *In vitro*: Mouse skin fibroblasts (MSF) — 2D, 3D spheroids, 3D ex vivo skin model; *In vivo*: Balb/c mice (male, 12 weeks) | No exogenous induction (normal cells/skin; assessed ECM synthesis promotion) | *In vitro*: 0.01, 0.1, 1, 10, 100 μg/mL (viability); 1, 10 μg/mL (migration); 1 μg/mL (3D/ECM); *In vivo*: RBE-containing cream (concentration not specified) | *In vitro*: 1 μg/mL; *In vivo*: not reported | *In vitro*: 24–48 h; 3 days (3D skin); *In vivo*: 14 or 28 days | Not reported | Untreated control (no RBE); blank cream only (*in vivo*) | (Yan et al., 2025) | **Partially compliant** |
| 75% ethanol extract of *Poria cocos* (Schw.) Wolf (Polyporaceae) sclerotium (PCE, Lipucan®); also tested 6 isolated lanostane triterpenoids | UPLC; isolated compounds identified by NMR and ESI-MS | *In vivo*: SD rats (male, 10 weeks); *In vitro*: Primary human dermal fibroblasts (HDFs) | *In vivo*: D-galactose (100 mg/kg/day, s.c.) for 8 weeks; *In vitro*: no exogenous induction | *In vivo* (oral): PCE 1, 3, 6 mg/kg; *In vitro* (6 compounds): 0.01, 0.1, 1 μM | *In vivo*: 6 mg/kg (collagen I), 3 mg/kg (HA); *In vitro*: 1 μM (most compounds) | *In vivo*: 4 weeks (daily oral); *In vitro*: 48 h | Not reported | Normal group (saline + distilled water); Control group (D-gal + distilled water) | (Chao et al., 2023) | **Partially compliant** |
| Water extract of dandelion (*Taraxacum officinale* F.H.Wigg. (Asteraceae)) — separate leaf, flower, root extracts | Not reported (no HPLC/LC-MS; only UV absorption spectra at 280–310 nm) | Human dermal fibroblasts (HDFs, ATCC) | UVB 200 mJ/cm² (60 s); H₂O₂ 100 μM (72 h, senescence model) | 30, 100, 300 μg/mL (tested 10–3000 μg/mL for cytotoxicity) | 30 μg/mL (leaf and flower extracts) | 30 min pretreatment + 24 h post-UVB; H₂O₂: 72 h co-treatment | Not reported | Untreated control (no UVB, no extract); UVB-only control | (Yang et al., 2015) | **Non-compliant** |
| 50% ethanol extract of *Prunella vulgaris* L. (Lamiaceae) spica (PVE) | HPLC (rosmarinic acid 1.49%, caffeic acid 0.33%, rutin 0.11%) | Normal human dermal fibroblasts (NHDFs) | UVB 144 mJ/cm² (peak 312 nm) | 1, 10, 100 μg/mL | 1 μg/mL (some markers); 100 μg/mL (maximal effect) | 24 h (ROS/mRNA); 72 h (MMP/IL-6 secretion); 1.5 h (signaling pathways) | Arbutin (DPPH assay only; no positive control in cell experiments) | Nonirradiated control; UVB-irradiated control | (Zhang et al., 2018) | **Partially compliant** |
| 50% ethanol extract of *Hibiscus mutabilis* L. (Malvaceae) (EHMF), purified by polyamide and macroporous resins | UPLC-MS/MS (22 active compounds: kaempferol, quercetin, naringenin, dihydroartemisinin, 4',7-Di-O-methylnaringenin, scrophulein, etc.) | *In vitro*: HaCaT; *In vivo*: ICR mice (male, 20–22 g) | *In vitro*: UVB 20 mJ/cm² + UVA 200 mJ/cm² (combined, 30 min); *In vivo*: UVB 20 mJ/cm² + UVA 200 mJ/cm² (48 h cumulative) | *In vitro*: EHMF-L 125 μg/mL, EHMF-H 500 μg/mL; *In vivo* (topical): EHMF-L 200 mg/kg, EHMF-H 400 mg/kg | *In vitro*: 125 μg/mL; *In vivo*: 200 mg/kg | *In vitro*: 2 h pretreatment + post-UV assessment; *In vivo*: 7 days (daily topical) | Methotrexate (MTX) — *in vitro*: 0.1 μg/mL; *in vivo*: 10 mg/kg topical | Control (no UV, no drug); Model (UV only); UV + Gel (carbomer gel vehicle) | (Chen et al., 2024) | **Compliant** |
| Aqueous extract of saffron (stigmas of *Crocus sativus* L. (Iridaceae)) | HPLC (crocin I 10.80%, crocin II 8.77%, picrocrocin 12.00%) | *In vivo*: C57BL/6J mice (female, 3-, 8-, 16-months-old) | Natural aging (no exogenous induction; 8- and 16-month-old vs. 3-month-old young controls) | 80 mg/kg (oral gavage) | 80 mg/kg (the only tested concentration) | 8 weeks (daily oral gavage) | Not reported | Age-matched control groups (distilled water) | (Xiao et al., 2024) | **Non-compliant** |
| 50% hydroethanolic extract of *Cordyceps sinensis* (Berk.) Sacc. (Cordycipitaceae) mycelium (CsEx) | UHPLC-DAD (cordycepin 6247 ± 20 μg/g, adenosine 470 ± 55 μg/g); Folin-Ciocalteu (polyphenols 21.2 mg GAE/g); Bradford (protein 23.6 mg BSA equiv/g); o-phthalaldehyde (peptides 9.4 mg/g); enzymatic assay (glucose 15.9, fructose 4.7, sucrose 1.4 mg/g) | *In vitro*: HaCaT, HDFs; Human: 40 healthy subjects (females, 40–65, Fitzpatrick I–III) | *In vitro* (ROS): H₂O₂ 450 μM; *In vitro* (others): no exogenous induction; Clinical: natural skin aging | *In vitro*: 0.0006% w/v, 0.002% w/v; Clinical: 6 mg/L (≈0.0006% w/v) topical, twice daily | *In vitro*: 0.0006% w/v; Clinical: 6 mg/L | *In vitro*: 6 h (sirtuin), 24 h (NAD⁺/ATP/collagen), 2 h (ROS); Clinical: 28 days | Resveratrol (100 μM), Ascorbic acid (500 μM), TGF-β (2.5 ng/mL) | Untreated cells (CTR); Placebo topical formulation | (Di Lorenzo et al., 2024) | **Compliant** |
| Water extract of *Panax ginseng* C.A.Mey. (Araliaceae) cv. Silvatica (forest ginseng) root, ethanol precipitation, D101 resin purification (FGE); also tested phenolic acid mixture (PAM) | HPLC-DAD (7 phenolic acids: p-hydroxybenzoic acid 2.72 g/100g, salicylic acid 0.80, vanillic acid 0.35, caffeic acid 0.25, chlorogenic acid 0.19, ferulic acid 0.18, protocatechuic acid 0.07) | HFF-1 cells (human foreskin fibroblasts) | UVA irradiation (1 J/cm²) | 6.25, 12.5, 25 μg/mL (FGE and PAM) | 6.25 μg/mL | 2 h pretreatment + 24 h post-UVA | N-Acetylcysteine amide (NAC) — concentration not specified | Untreated control (no UVA, no drug); UVA-irradiated control | (Sun et al., 2023) | **Compliant** |
| Water decoction of *Kochiae Fructus* (KF, dried fruits of *Kochia scoparia* (L.) Schrad. (Amaranthaceae)); also tested CHF, EAF, NBF, PWF fractions | Total phenolics (Folin-Ciocalteu): EAF 112.90 mg GAE/g; Total flavonoids (AlCl₃): EAF 329.60 mg RE/g (no HPLC/LC-MS individual compound identification) | *In vitro* (cell-free): pBR322 DNA oxidation, BSA protein oxidation; *In vivo* (toxicity): KM mice (acute), SD rats (subacute) | DNA/protein oxidation: AAPH (cell-free chemical system); No skin aging model | Antioxidant: concentration gradients (IC₅₀); Acute toxicity: 15 g/kg BW; Subacute: 2.5, 5.0, 10.0 g/kg BW | DPPH IC₅₀: KFW 29.55 μg/mL, EAF 60.56, NBF 30.12; No skin aging efficacy | Acute: 14 days observation; Subacute: 28 days daily oral | Ascorbic acid (DPPH only) | Distilled water (*in vivo*); DMSO/PBS blanks (antioxidant assays) | (Chen et al., 2022) | **Non-compliant** |
| Resina Draconis (RD, resin from *Dracaena cochinchinensis* (Lour.) S.C.Chen (Asparagaceae)) — RD-loaded polymeric micelle (RDPM) hydrogel | LC-QqQ-MS/MS (5 constituents: loureirin A, loureirin B, 7,4'-dihydroxyflavone, resveratrol, pterostilbene); EE >90% (except LB 80.6%); DL 0.038–0.116% | *In vitro*: Human dermal fibroblasts (UV-induced senescence, Supplementary Fig. S4 only); *In vivo*: SKH-1 hairless mice (female, 4–6 weeks) | *In vivo*: UVA+UVB combined, 3×/week, 15 weeks (1→4 MED); *In vitro*: UV (details in Supplementary) | *In vivo* (topical): 0.25% RD, 1.0% RD; *In vitro*: not reported | *In vivo*: 0.25% RDPM hydrogel | *In vivo*: 15 weeks (daily topical); *In vitro*: not reported | Not reported | Normal (no UV, no treatment); Model (UV only); Blank hydrogel (UV + drug-free gel) | (Liu et al., 2026) | **Partially compliant** |
| Aeroponic root extract of *Morus alba* L. (Moraceae) (nitrogen-deprived, N−); also tested isolated prenylated flavonoids | UHPLC-MS + NMR (5 prenylated flavonoids: moracenin B, kuwanon C, moracenin A, wittiorumin F, morusin); nitrogen deprivation increased total marker content 18-fold vs. commercial extract | *In vitro*: NHDFs (ATCC CRL-2522); Human: 22 Caucasian females (45–70 years), randomized double-blind placebo-controlled, split-face | *In vitro*: UVB 125 mJ/cm², once daily ×4 days + 72 h recovery; Clinical: natural skin aging (crow's feet) | *In vitro* (gene): 0.2%; *In vitro* (CICP/UVB): 0.00625%, 0.0125%; Clinical: 1% in cream, twice daily ×8 weeks | *In vitro*: 0.00625%; Clinical: 1% | *In vitro*: 24 h pretreatment + 4-day UVB + 72 h recovery; Clinical: 8 weeks | TGF-β1 (20 ng/mL) for gene expression; SDS (0.008%) for MTS validation; No positive control for collagenase/clinical | Untreated/vehicle control; Placebo cream | (Chajra et al., 2020) | **Compliant** |
| Water extract of pearl (from freshwater pearl oyster *Hyriopsis cumingii*), RTSES extraction | Contains 10 μg/mL protein, 90–140 ppm amino acids, 3200 ppb Ca²⁺, 339 ppb Mg²⁺ (no HPLC/LC-MS fingerprinting) | Hs68 cells (fibroblast line); HFFs (human primary fibroblasts) | No exogenous aging/damage induction — scratch wound healing assay (mechanical wound) | 300 μg/mL (the only tested concentration) | 300 μg/mL (the only tested concentration; no dose gradient tested) | Scratch: 16 h; RT-qPCR: 1 day; Observation: up to 7 days | Not reported | PL-free DMEM medium (10% FBS) | (Li et al., 2013) | **Non-compliant** |
| Water extract of pearl (from *Pinctada fucata martensii* Dunker), formulated as pearl extract gel (PEG) | HPLC with PITC derivatization (14 amino acids identified: asparagine, glutamic acid, serine, glycine, arginine, threonine, alanine, proline, valine, isoleucine, leucine, phenylalanine, tryptophan, lysine) | *In vivo*: KM mice (female, 6-week-old) | UVA+UVB combined, 5×/week ×9 weeks; start 100 mJ/cm² → 4 MED by week 4, total ~13 J/cm² | Topical: PEG-L 0.2, PEG-M 0.4, PEG-H 0.8 mg/mouse (applied 2 h before UV) | 0.2 mg/mouse (lowest tested, effective) | 9 weeks (5×/week topical, 2 h before UV) | Vitamin E (VE) — 0.8 mg/mouse topical | NC (no UV, no treatment); SC (shaved only); MC (UV only); VC (UV + blank gel) | (Zhang et al., 2022) | **Compliant** |

**Table Notes:**

**Compliance classification definitions (based on Frontiers in Pharmacology Four Pillars guidelines, Heinrich et al., 2020):**

- **Compliant** – The study meets all core pharmacological and composition requirements: full chemical characterization (ConPhyMP-compliant), multiple dose/concentration testing (dose gradient), validated experimental models (*in vitro*/*in vivo*/clinical as appropriate), and presence of both positive and negative controls for the primary efficacy endpoints.
- **Partially compliant** – The study meets most core requirements but has one or more of the following limitations: missing positive control for the primary efficacy endpoint, incomplete reporting of minimum effective dose or *in vivo* concentration, or absence of quantitative marker compound data when only qualitative identification is provided.
- **Non-compliant** – The study fails to meet one or more fundamental requirements: no HPLC/LC-MS chemical profiling (fails ConPhyMP), single-dose only without dose gradient, no positive control for efficacy assessment, or reliance solely on cell-free chemical assays (e.g., DPPH/ABTS/FRAP) as pharmacological evidence without therapeutically relevant models.

**Abbreviations:** “NR” indicates that the information was not reported in the original study.

**Supplementary Table 2.** Composition and preparation of polyherbal formulas included in this review.

| **Formula name** | **Species included (with authority and family)** | **Botanical drug part used** | **Composition / ratio** | **Extraction / preparation method** | **Quality control** | **Ref.** | **Four Pillars Compliance & Limitations** |
| --- | --- | --- | --- | --- | --- | --- | --- |
| Erzhi Formula | *Ligustrum lucidum* W.T. Aiton (Oleaceae); *Eclipta prostrata* Lour. (Asteraceae) | Fruit; aerial part | NR | 80% ethanol reflux, 10×, 2×, 2 h each, concentrated, vacuum-dried | UPLC: 7 components quantified (salidroside, echinacoside, isoquercitrin, verbascoside, isochlorogenic acid A, specnuezhenide, wedelolactone) | (Liu et al., 2023) | **Partially compliant** |
| Modified Qing'e Formula | *Eucommia ulmoides* Oliv. (Eucommiaceae); *Psoralea corylifolia* L. (Fabaceae); *Salvia miltiorrhiza* Bunge (Lamiaceae) | NR | 2:1:2 | 75% ethanol reflux, 10×, 2×, filtered hot, rotary evaporated, vacuum freeze-dried 24 h | UHPLC-Q-TOF-MS: 19 compounds tentatively identified (qualitative only) | (Zhu et al., 2022) | **Partially compliant** |
| Danggui Buxue Decoction | *Angelica sinensis* (Oliv.) Diels (Apiaceae); *Astragalus membranaceus* (Fisch.) Bunge (Fabaceae) | NR | 1:5 | NR (commercial lyophilized powder; 5.45 g raw herb per 1 g powder) | NR | (Chen et al., 2026) | **Non-compliant** |
| Sijunzi Tang | *Panax ginseng* C.A.Mey. (Araliaceae); *Atractylodes macrocephala* Koidz. (Asteraceae); *Poria cocos* (Schw.) Wolf (Polyporaceae); *Glycyrrhiza uralensis* Fisch. ex DC. (Fabaceae) | Root; rhizome; sclerotium; root | 1:1:1:1 | 95% ethanol soak 1 h, ultrasonic 40°C 1.5 h, filtered, evaporated, water suspension, ethyl acetate extraction, fraction vacuum-evaporated (yield 2.7%) | UPLC-QTOF-MS/MS: 98 compounds; key compounds confirmed | (Ke et al., 2024) | **Compliant** |
| Qibao Meiran Dan | *Poria cocos* (Schw.) Wolf (Polyporaceae); *Angelica sinensis* (Oliv.) Diels (Apiaceae); *Achyranthes bidentata* Blume (Amaranthaceae); *Cuscuta chinensis* Lam. (Convolvulaceae); *Lycium barbarum* L. (Solanaceae); *Reynoutria multiflora* (Thunb.) Moldenke (Polygonaceae); *Psoralea corylifolia* L. (Fabaceae) | NR | NR | NR | Network pharmacology: 72 active compounds predicted via TCMSP/TCMID; no HPLC/UPLC data | (Ke et al., 2022) | **Non-compliant** |
| Yang Yan Qing E Wan | *Eucommia ulmoides* Oliv. (Eucommiaceae); *Psoralea corylifolia* L. (Fabaceae); *Reynoutria multiflora* (Thunb.) Moldenke (Polygonaceae); *Astragalus complanatus* [R.Br](https://r.br/). (Fabaceae); *Juglans mandshurica* Maxim. (Juglandaceae) | NR | 1:1:1:1:1 | NR (concentrated to 1.3 g crude drug/mL) | NR | (Tian et al., 2020) | **Non-compliant** |
| Anti-aging Formula | *Rehmannia glutinosa* (Gaertn.) DC. (Orobanchaceae); *Panax ginseng* C.A.Mey. (Araliaceae); *Asparagus cochinchinensis* (Lour.) Merr. (Asparagaceae); *Ophiopogon japonicus* (Thunb.) Ker Gawl. (Asparagaceae); *Lycium barbarum* L. (Solanaceae); *Poria cocos* (Schw.) Wolf (Polyporaceae) | NR | 409:167:26:26:26:77 | NR (patent CN1943707B) | UHPLC-Q-TOF-MS/MS: 113 compounds tentatively identified (qualitative only) | (Zhu et al., 2024) | **Partially compliant** |
| Anshen Bunao Syrup | *Cervus nippon* Temminck (Cervidae); *Reynoutria multiflora* (Thunb.) Moldenke (Polygonaceae); *Epimedium brevicornu* Maxim. (Berberidaceae); *Zingiber officinale* Rosc. (Zingiberaceae); *Glycyrrhiza uralensis* Fisch. ex DC. (Fabaceae); *Ziziphus jujuba* Mill. (Rhamnaceae); plus Vitamin B1 | NR | NR | NR (commercial syrup) | UPLC-Q-TOF-MS: 159 compounds identified (qualitative only) | (Sun et al., 2026) | **Non-compliant: contains animal-derived material (*Cervus nippon*** Temminck (Cervidae)**) without ethical/sustainable sourcing evidence** |
| Ba Zhen Tang | *Panax ginseng* C.A.Mey. (Araliaceae); *Atractylodes macrocephala* Koidz. (Asteraceae); *Poria cocos* (Schw.) Wolf (Polyporaceae); *Angelica sinensis* (Oliv.) Diels (Apiaceae); *Ligusticum striatum* DC. (Apiaceae); *Paeonia lactiflora* Pall. (Paeoniaceae); *Rehmannia glutinosa* (Gaertn.) DC. (Orobanchaceae); *Glycyrrhiza uralensis* Fisch. ex DC. (Fabaceae) | Root; rhizome; sclerotium; root; rhizome; root; root; root | 1:1:1:1:1:1:1:1 | Water decoction, concentrated to 1.3 g crude drug/mL | NR (compounds predicted via TCMSP; no HPLC/UPLC data) | (Han et al., 2022) | **Partially compliant** |
| Five-Colored Plants Combination | *Vigna radiata* (L.) R.Wilczek (Fabaceae); *Tuber magnatum* Pico (Tuberaceae); *Tuber melanosporum* Vittad. (Tuberaceae); *Chrysanthemum × morifolium* (Ramat.) Hemsl. (Asteraceae); *Lycium barbarum* L. (Solanaceae) | Seed; fruiting body; fruiting body; flower bud; fruit | Black truffle 35%, white truffle 35%, goji berry 10%, chrysanthemum 10%, mung bean 10% | NR | Network pharmacology: 27 core compounds predicted; no HPLC/UPLC data | (Tao et al., 2025) | **Non-compliant:** |
| MP Gel | *Cnidium monnieri* (L.) Cusson (Apiaceae); *Arnebia euchroma* (Royle ex Benth.) I.M.Johnst. (Boraginaceae); *Angelica sinensis* (Oliv.) Diels (Apiaceae); *Poria cocos* (Schw.) Wolf (Polyporaceae); *Borneolum* (resin, *Cinnamomum camphora*) | Fruit; root; root; sclerotium; resin | NR | Mixed extracts + probiotic fermentation supernatant, carbomer gel | Network pharmacology: 63 active compounds incl. β-sitosterol, stigmasterol, shikonin; no HPLC/UPLC data | (Xu et al., 2023) | **Non-compliant** |
| Wan'an Wan | *Dioscorea polystachya* Turcz. (Dioscoreaceae); *Cuscuta chinensis* Lam. (Convolvulaceae); *Cornus officinalis* Siebold & Zucc. (Cornaceae); *Schisandra chinensis* (Turcz.) Baill. (Schisandraceae); *Eucommia ulmoides* Oliv. (Eucommiaceae); *Achyranthes bidentata* Blume (Amaranthaceae); *Poria cocos* (Schw.) Wolf (Polyporaceae); *Alisma orientale* (Sam.) Juzep. (Alismataceae) | Rhizome; seed; fruit pulp; fruit; bark; root; sclerotium; rhizome | M3 selected (sovereign: D. polystachya 9.0 + C. chinensis 6.0; minister: C. officinalis 9.0 + S. chinensis 9.0; assistant: E. ulmoides 4.5 + A. bidentata 9.0; courier: P. cocos 9.0 + A. orientalis 6.0) | 30% 1,3-butanediol, ultrasound-assisted, optimized per herb (liquid ratio 1:20–1:30, ultrasonic time 50–80 min, 2–3×, 50°C) | HPLC: morroniside, loganin, schisandrin A, pinoresinol diglucoside, β-ecdysterone confirmed | (Lin et al., 2025) | **Compliant** |
| Five-herb Combination | *Panax ginseng* C.A.Mey. (Araliaceae); *Polygonatum cyrtonema* Hua (Asparagaceae); *Epiphyllum oxypetalum* (DC.) Haw. (Cactaceae); *Nelumbo nucifera* Gaertn. (Nelumbonaceae); *Osmanthus fragrans* Lour. (Oleaceae) | Root; rhizome/root; flower; aerial parts; flower | NR | P. ginseng, P. cyrtonema, E. oxypetalum: per Zhao et al., 2024; N. nucifera: Zeta Fraction technology (US Patent 7,442,391 B2); O. fragrans: NR | HPLC-MS/NMR: ginsenosides Rg1/Rb1/Re, kaempferol, liriodendrin, quercetin-3-O-β-D-galactoside, caffeic acid (qualitative) | (Feng et al., 2024) | **Partially compliant** |
| Bazi Bushen Capsule | *Cuscuta chinensis* Lam. (Convolvulaceae); *Lycium barbarum* L. (Solanaceae); *Schisandra chinensis* (Turcz.) Baill. (Schisandraceae); *Cnidium monnieri* (L.) Cusson (Apiaceae); *Rosa laevigata* Michx. (Rosaceae); *Rubus chingii* Hu (Rosaceae); *Allium tuberosum* Rottler ex Spreng. (Amaryllidaceae); *Melia toosendan* Siebold & Zucc. (Meliaceae); *Epimedium brevicornu* Maxim. (Berberidaceae); *Morinda officinalis* [F.C.How](https://f.c.how/) (Rubiaceae); *Cistanche deserticola* [Y.C.Ma](https://y.c.ma/) (Orobanchaceae); *Rehmannia glutinosa* (Gaertn.) DC. (Orobanchaceae); *Cyathula officinalis* K.C.Kuan (Amaranthaceae); *Panax ginseng* C.A.Mey. (Araliaceae); *Cervus nippon* Temminck (Cervidae); *Hippocampus* sp. (Syngnathidae) | Seed; fruit; fruit; fruit; fruit; fruit; seed; fruit; aerial part; root; stem; root; root; root; antler; whole animal | NR | NR (commercial capsule; water/ethanol extraction per manufacturer) | UPLC fingerprint: 14 characteristic peaks incl. neochlorogenic acid, chlorogenic acid, cryptochlorogenic acid, isoquercitrin, hyperoside, verbascoside, epimedin A, icariin, baohuoside I, imperatorin, osthole, catalpol, deoxyschizandrin, schisandrin B (RSD <2%) (Huang et al., 2020, 2021) | (Jiang et al., 2025) | **Non-compliant:** **contains animal-derived materials (*Cervus nippon*** Temminck (Cervidae)**, *Hippocampus* sp.** (Syngnathidae)**) without ethical/sustainable sourcing evidence** |
| Bazi Bushen Capsule | *Cuscuta chinensis* Lam. (Convolvulaceae); *Lycium barbarum* L. (Solanaceae); *Epimedium brevicornu* Maxim. (Berberidaceae); *Schisandra chinensis* (Turcz.) Baill. (Schisandraceae); *Cnidium monnieri* (L.) Cusson (Apiaceae); *Rosa laevigata* Michx. (Rosaceae); *Rubus chingii* Hu (Rosaceae); *Allium tuberosum* Rottler ex Spreng. (Amaryllidaceae); *Morinda officinalis* [F.C.How](https://f.c.how/) (Rubiaceae); *Cistanche deserticola* [Y.C.Ma](https://y.c.ma/) (Orobanchaceae); *Rehmannia glutinosa* (Gaertn.) DC. (Orobanchaceae); *Cyathula officinalis* K.C.Kuan (Amaranthaceae); *Panax ginseng* C.A.Mey. (Araliaceae); *Cervus nippon* Temminck (Cervidae); *Hippocampus* sp. (Syngnathidae); *Melia toosendan* Siebold & Zucc. (Meliaceae) | Seed; fruit; fruit; fruit; fruit; fruit; seed; root; stem; root; root; root; antler; whole animal; fruit | NR | NR (commercial capsule) | UPLC fingerprint reported in Huang et al., 2020, 2021 (14 characteristic peaks); no quantitative data in this study | (Xu et al., 2023) | **Non-compliant:** **contains animal-derived materials (*Cervus nippon*, *Hippocampus* sp.** (Syngnathidae)**) without ethical/sustainable sourcing evidenc** |
| Bazi Bushen Capsule | *Cyathula officinalis* K.C.Kuan (Amaranthaceae); *Rubus chingii* Hu (Rosaceae); *Lycium barbarum* L. (Solanaceae); *Rosa laevigata* Michx. (Rosaceae); *Cuscuta chinensis* Lam. (Convolvulaceae); *Epimedium brevicornu* Maxim. (Berberidaceae); *Cistanche deserticola* [Y.C.Ma](https://y.c.ma/) (Orobanchaceae); *Melia toosendan* Siebold & Zucc. (Meliaceae); *Panax ginseng* C.A.Mey. (Araliaceae); *Schisandra chinensis* (Turcz.) Baill. (Schisandraceae); *Cnidium monnieri* (L.) Cusson (Apiaceae); *Morinda officinalis* [F.C.How](https://f.c.how/) (Rubiaceae); *Rehmannia glutinosa* (Gaertn.) DC. (Orobanchaceae) | Root; fruit; fruit; fruit; seed; aerial part; stem; fruit; root; fruit; fruit; root; root | NR | NR (commercial capsule) | Network pharmacology: 12 key compounds incl. quercetin, kaempferol, arachidonate, suchilactone, ammidin, deoxyharringtonine, sitosterol, mandenol, ethyl linolenate, stigmasterol, poriferast-5-en-3beta-ol, cholesterol (no HPLC/UPLC data; cites Huang et al., 2023 for UPLC fingerprint) | (Zhao et al., 2025) | **Non-compliant:** **contains animal-derived material (*Cervus nippon*) without ethical/sustainable sourcing evidence** |

**Table Notes:**

**Compliance classification definitions (based on Frontiers in Pharmacology Four Pillars guidelines, Heinrich et al., 2020):**

- **Compliant** – The study provides complete botanical information (all species with authority and family, drug part used), clear quantitative composition/ratio, full extraction procedure, and experimental chemical profiling with quantitative marker data or validated fingerprinting.
- **Partially compliant** – The study meets some but not all composition requirements. Typical limitations: missing botanical drug part, incomplete extraction details, or qualitative-only chemical identification without quantitative marker content.
- **Non-compliant** – The study fails to provide essential composition information (e.g., no extraction procedure, no quality control data, only in silico predictions without experimental chemical profiling) or **contains animal-derived materials without ethical/sustainable sourcing evidence**, which violates the Four Pillars ethical requirements.

**Abbreviations:** “NR” indicates that the information was not reported in the original study.

**Supplementary Table 3.** Pharmacological parameters of formula studies

| **Formula name** | **Model type** | **Induction method** | **Dose / Concentration range** | **Minimum effective concentration / dose** | **Treatment duration** | **Positive control** | **Negative control** | **Ref.** | **Four Pillars Compliance & Limitations** |
| --- | --- | --- | --- | --- | --- | --- | --- | --- | --- |
| ErZhiFormula (EZF) | *In vitro*: HaCaT, 293T; *In vivo*: ICR mice (male, 4 weeks) | *In vitro*: UVB 60 mJ/cm²; *In vivo*: UVA+UVB combined, 5×/week ×9 weeks (UVA total 94.52 J/cm², UVB total 9.45 J/cm²) | *In vitro*: 12.5–200 μg/mL; *In vivo* (topical): EZF low 0.67 mg/g, high 1.33 mg/g | *In vitro*: 12.5 μg/mL; *In vivo*: 0.67 mg/g | *In vitro*: 24 h; *In vivo*: 9 weeks | tBHQ (ARE assay only); ATRA (Nrf2 inhibitor, not efficacy positive control) | *In vitro*: Control (no UVB, no EZF), Model (UVB only); *In vivo*: Control (no UV), Model (UV only), Base (UV + blank gel) | (Liu et al., 2023) | **Partially compliant** |
| Modified Qing'e Formula (MQEF) | *In vitro*: HaCaT, HEK293T; *In vivo*: ICR mice (male, 18–22 g) | *In vitro*: UVB 60 mJ/cm²; *In vivo*: UVA+UVB combined, 5×/week ×9 weeks (UVB total 9.45 J/cm², UVA total 94.5 J/cm²) | *In vitro*: 1, 10, 100 μg/mL; *In vivo* (topical): MQEF low 0.67 mg/g, high 1.33 mg/g | *In vitro*: 1 μg/mL; *In vivo*: 0.67 mg/g | *In vitro*: 24 h; *In vivo*: 9 weeks | Not reported | *In vitro*: Control (no UVB, no MQEF), Model (UVB only); *In vivo*: Control (no UV), Model (UV only), Base (UV + blank gel) | (Zhu et al., 2022) | **Partially compliant** |
| Danggui Buxue Decoction (DBD) | *In vitro*: HaCaT cells | H₂O₂ (600 μmol/L, 1 h) | 400, 800, 1600 μg/mL (24 h pretreatment); optimal 400 μg/mL | 400 μg/mL | 24 h pretreatment + 1 h H₂O₂ | Not reported | Blank control (no H₂O₂, no DBD); Model (H₂O₂ only) | (Chen et al., 2026) | **Partially compliant** |
| Si Jun Zi Tang (SJZT) | *In vitro*: Rat EpiSCs; *In vivo*: C57BL/6J mice (male, 4–6 weeks) | *In vitro*: UVB 5 or 10 mJ/cm²; *In vivo*: UVB 6 weeks (60→120→180 mJ/cm²) | *In vitro*: 15.6, 31.3, 62.5 μg/mL; *In vivo* (topical): SJZT 0.5%, 2% | *In vitro*: 15.6 μg/mL; *In vivo*: 0.5% | *In vitro*: 24 h; *In vivo*: 6 weeks | Fisetin — *in vitro*: 2 μM; *in vivo*: 0.5% topical | *In vitro*: Control (no UVB), Model (UVB only); *In vivo*: Control (no UV), Model (UV only), Solvent (UV + vehicle) | (Ke et al., 2024) | **Compliant** |
| Qi-Bao-Mei-Ran-Dan (QBMRD) | *In vitro*: NHSFs (primary human skin fibroblasts) | H₂O₂ (150 μmol/L, 2 h) | QBMRD-containing serum (concentration not specified in article) | Not reported | Not clearly specified | Not reported | Normal group (no H₂O₂, no QBMRD); Blank group; Model group (H₂O₂ only) | (Li et al., 2022) | **Non-compliant** |
| Qibao Meiran Dan (QBMRD) | *In vitro*: NHSFs (primary cells); *In vivo* (serum preparation): SD rats (female, 4 months) | H₂O₂ (150 μmol/L, 2 h) | *In vitro*: 10% QBMRD-containing serum; *In vivo* (serum prep): 3.1 g/kg QBMRD (oral, 7 days) | 10% QBMRD-containing serum (the only tested concentration) | Cell culture: 24 h; Serum prep: 7 days | Not reported | Normal group; Blank group; Model group (H₂O₂ only) | (Ke et al., 2022) | **Non-compliant** |
| Yang Yan Qing E Wan (YYQEW) | *In vitro*: NHSFs (primary cells); *In vivo* (serum preparation): SD rats (female, 4 months) | H₂O₂ (150 μmol/L, 2 h) | *In vitro*: 10% YYQEW-containing serum; *In vivo* (serum prep): 6.173 g/kg YYQEW (oral, 7 days) | 10% YYQEW-containing serum (the only tested concentration) | Cell culture: 24 h; Serum prep: 7 days | Not reported | Normal group; Blank group; Model group (H₂O₂ only) | (Tian et al., 2020) | **Non-compliant** |
| Anti-aging formula (AAF) | *In vitro*: HSF cells; *In vivo*: ICR mice (4 weeks, 18–20 g) | *In vitro*: AAPH (4 mM, 24 h); *In vivo*: UVA+UVB combined, 5×/week ×9 weeks (UVB total 9.45 J/cm², UVA total 94.5 J/cm²) | *In vitro*: 100, 200, 500 μg/mL; *In vivo* (oral): AAF-L 190.89 mg/kg, AAF-H 381.78 mg/kg | *In vitro*: 200 μg/mL; *In vivo*: 190.89 mg/kg | *In vitro*: 24 h; *In vivo*: 9 weeks | Vitamin C — *in vivo*: 300 mg/kg (oral) | *In vitro*: Control (no AAPH), Model (AAPH only); *In vivo*: Control (no UV), Model (UV only) | (Zhu et al., 2024) | **Compliant** |
| Anshen Bunao Syrup (ABS) | *In vivo*: SD rats (male, 8–10 weeks) | D-Galactose (120 mg/kg/d, s.c.) for 8 weeks | Oral: ABS-L 1.05, ABS-M 2.10, ABS-H 4.20 mL/kg/d | ABS-L: 1.05 mL/kg/d | 2 weeks pretreatment + 8 weeks co-administration (total 10 weeks) | Rapamycin — 0.5 mg/kg/d (oral) | CON (saline + water); MOD (D-gal + water) | (Sun et al., 2026) | **Compliant** |
| Ba Zhen Tang (BZT) | *In vitro*: HaCaT cells; *In vivo* (serum preparation): SD rats (male, 6–8 months) | UVB (20 mJ/cm², once daily ×5 days) | *In vitro*: 10% BZT-containing serum; *In vivo* (serum prep): 2.5 g/kg BZT (oral, 7 days) | 10% BZT-containing serum (the only tested concentration) | 24 h pretreatment + 5-day UVB | Not reported | Control (UVB + control serum, no BZT) | (Han et al., 2022) | **Non-compliant** |
| Five-colored plant combination | *In vitro*: 3T3 fibroblasts, human dermal fibroblasts; *Ex vivo*: Human skin explants | *In vitro* (SA-β-gal): H₂O₂; *Ex vivo*: UVA (30 J/cm²) + UVB (50 mJ/cm²) ×4 days | *In vitro*: 0.5% (v/v); *Ex vivo*: 2 μL topical | 0.5% (the only tested concentration) | *In vitro*: 24 h; *Ex vivo*: 7 days (4d irradiation+3d recovery) | *In vitro* (scratch): serum-free medium; *Ex vivo*: VC (100 μg/mL) + VE (7 μg/mL) | *In vitro*: Basal medium + H₂O₂; *Ex vivo*: UVA+UVB irradiated untreated | (Tao et al., 2025) | **Partially compliant** |
| MP gel | *In vivo*: Kunming mice (female, 4 weeks, 18–20 g) | UVA+UVB combined, 4×/week ×8 weeks; 1 MED (198 mJ/cm²) → 4 MED by week 4 | Topical: MP gel (exact concentrations not specified); Blank gel (without 5 extracts) | Not reported | 4 weeks (weeks 5–8, applied 0.5 h after UV) | 0.1% Tretinoin (retinoic acid) — topical | NC (no UV); MC (UV only) | (Xu et al., 2023) | **Partially compliant** |
| WAW formula | *In vitro*: Human skin fibroblasts / HFF-1 | MGO (methylglyoxal, 4 mM, 48 h) — AGE-induced senescence | 0.5%, 1%, 2% (M3 optimal); M1, M2, M3 ratios tested | 0.5% (M3, lowest tested, effective) | 48 h (co-treatment with MGO) | L-Carnosine (concentration not specified) | Control (no MGO, no M3); MGO model (MGO only) | (Lin et al., 2025) | **Compliant** |
| 5 TCM combination | *In vitro*: NHDFs | H₂O₂ (400 μM, 2h ×3 + 5 passages) — replicative senescence + oxidative stress | 1 TCM (0.5%), 3 TCM (total 0.5%), 5 TCM (each 0.1%, total 0.5%) | 5 TCM (best efficacy, total 0.5%) | 24 h per H₂O₂ cycle ×3 + 5 passages | TGF-β1 (100 ng/mL) | Untreated cells; H₂O₂-stressed cells | (Feng et al., 2024) | **Compliant** |
| Bazi Bushen capsule (BZBS) | *In vivo*: Lgr5-EGFP mice (male, 3–4 months); *In vitro*: RDFs, HFSCs, ARE-luc iBMDM | *In vivo*: UVA+UVB combined, 4 weeks; *In vitro*: UVA (~5.6 J/cm², 10 min) | *In vivo* (oral): BZBS 1, 2 g/kg/d; *In vitro* (BZBS): 50, 100 μg/mL; Verbascoside/epimedin A: 50, 100 μM | *In vivo*: 1 g/kg; *In vitro*: 50 μg/mL (BZBS), 50 μM (compounds) | *In vivo*: 4 weeks; *In vitro*: 24 h post-UVA | NMN — 200 mg/kg/d (oral, *in vivo*) | *In vivo*: Control (no UV), Model (UV + vehicle); *In vitro*: Control (no UVA), Model (UVA only) | (Jiang et al., 2025) | **Compliant** |
| Bazi Bushen capsule (BZBS) | *In vivo*: SAMP6 mice (male, 9–10 weeks, 25–35 g) | Natural aging (SAMP6 senescence-accelerated model; no exogenous induction) | 2.8 g/kg/d (oral gavage) | 2.8 g/kg/d (the only tested concentration) | 9 weeks (daily oral) | Not reported | SAMP6 control group (vehicle only) | (Xu et al., 2023) | **Non-compliant** |
| Bazi Bushen capsule (BZBS) | *In silico*: Network pharmacology; *Clinical*: 35 female volunteers (aged 30–50, skin laxity) | Natural skin laxity (no exogenous induction; volunteers with skin laxity ≥6 months) | 2 capsules (0.4 g/capsule), 3×/day (total 2.4 g/day), oral, 12 weeks | 2.4 g/day (the only tested concentration) | 12 weeks | Not reported (single-arm trial) | Baseline (0 week) vs. post-treatment (12 weeks) within-group comparison | (Zhao et al., 2025) | **Non-compliant** |

**Table Notes:**

**Compliance classification definitions (based on Frontiers in Pharmacology Four Pillars guidelines, Heinrich et al., 2020):**

- **Compliant** – The study meets all core pharmacological requirements: multiple dose/concentration testing (full dose gradient), validated experimental models (*in vitro*/*in vivo*/clinical as appropriate), and presence of both positive and negative controls for the primary efficacy endpoints.
- **Partially compliant** – The study meets some but not all pharmacological requirements. Typical limitations: single-dose testing (no dose gradient), absence of positive control, serum pharmacology without direct extract treatment, or incomplete reporting of dose/concentration parameters.
- **Non-compliant** – The study fails to meet one or more fundamental pharmacological requirements: single-dose only with no justification, no positive control for efficacy assessment, serum pharmacology without direct extract treatment (unless justified as an early-stage exploratory study), no negative control, or clinical studies without a control group.

**Abbreviations:** “NR” indicates that the information was not reported in the original study.
